# Earlier second polar body transfer and further mitochondrial carryover removal for potential mitochondrial replacement therapy

**DOI:** 10.1002/mco2.217

**Published:** 2023-05-08

**Authors:** Wenzhi Li, Xiaoyu Liao, Kaibo Lin, Renfei Cai, Haiyan Guo, Meng Ma, Yao Wang, Yating Xie, Shaozhen Zhang, Zhiguang Yan, Jiqiang Si, Hongyuan Gao, Leiwen Zhao, Li Chen, Weina Yu, Chen Chen, Yun Wang, Yanping Kuang, Qifeng Lyu

**Affiliations:** ^1^ Department of Assisted Reproduction, Shanghai Ninth People's Hospital Shanghai Jiao Tong University School of Medicine Shanghai China

**Keywords:** assisted reproductive technology, mitochondrial disease inheritance, mitochondrial genetic drift, mitochondrial replacement therapy, second polar body transfer

## Abstract

The second polar body (PB2) transfer in assisted reproductive technology is regarded as the most promising mitochondrial replacement scheme for preventing the mitochondrial disease inheritance owing to its less mitochondrial carryover and stronger operability. However, the mitochondrial carryover was still detectable in the reconstructed oocyte in conventional second polar body transfer scheme. Moreover, the delayed operating time would increase the second polar body DNA damage. In this study, we established a spindle‐protrusion‐retained second polar body separation technique, which allowed us to perform earlier second polar body transfer to avoid DNA damage accumulation. We could also locate the fusion site after the transfer through the spindle protrusion. Then, we further eliminated the mitochondrial carryover in the reconstructed oocytes through a physically based residue removal method. The results showed that our scheme could produce a nearly normal proportion of normal‐karyotype blastocysts with further reduced mitochondrial carryover, both in mice and humans. Additionally, we also obtained mouse embryonic stem cells and healthy live‐born mice with almost undetectable mitochondrial carryover. These findings indicate that our improvement in the second polar body transfer is conducive to the development and further mitochondria carryover elimination of reconstructed embryos, which provides a valuable choice for future clinical applications of mitochondrial replacement.

## Graphical abstract

We established an optimized second polar transfer scheme in mitochondrial replacement therapy. This enables earlier transfer of the second polar body to avoid the accumulation of DNA damage in the second polar body. And, by retaining the spindle protrusion on the second polar body, we accurately located the site where the second polar body was incorporated into the enucleated oocyte. Then we performed a physically based residue method to further eliminate mitochondrial carryover.

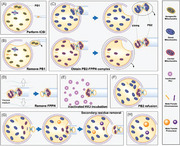

## 1 INTRODUCTION

Mitochondrial disease is a maternally inherited genetic disease caused by mitochondrial DNA (mtDNA) mutations, many of which are lethal or disabling.^1^, ^2^, ^3^ The mtDNA mutations will accumulate in cells with the increasing age of the carriers, which will lead to energy metabolism disorders, including abnormalities in respiratory chain, reduced adenosine triphosphate synthesis, and increased reactive oxygen species production, and eventually induce diabetes, neurodegenerative diseases, and cardiovascular‐related diseases.^1^, ^3^, ^4^, ^5^, ^6^ However, due to the complexity of the body, it is almost impossible to cure such diseases in adults.^1^, ^2^, ^3^, ^7^, ^8^ Therefore, if mitochondria with pathogenic genes can be removed or replaced in germ cells or early embryos through assisted reproductive technology, this will effectively block the inheritance of such diseases in offspring.^9^

In recent years, a new therapeutic strategy, mitochondrial replacement (MR), has been used to transfer the nuclear genetic materials (spindle [SP], first/second polar body [PB1/PB2], and pronucleus) of oocytes with pathogenic mitochondria into a healthy enucleated oocyte (reconstructed oocyte/zygote).^9^, ^10^ Under this strategy, only a small number of pathogenic mitochondrial carryover are carried into the normal enucleated oocytes during the transfer of the nuclear genetic material. Therefore, theoretically, as the level of the mutated mitochondria is reduced, the quality of life of offspring obtained by this strategy should be improved. At present, relevant research and clinical pre‐experiments have been widely carried out in mice, primates, and humans.^11^, ^12^, ^13^, ^14^, ^15^, ^16^, ^17^, ^18^ However, its clinical application has rarely been reported even though the first human baby produced by MR was born many years ago.^14^ There are still some problems in MR related to healthy offspring that should be solved.

The most important problem is the risk of mitochondrial genetic drift.^19^, ^20^, ^21^, ^22^, ^23^ In embryonic stem (ES) cells isolated from human MR reconstructed embryos, even though the initial mtDNA carryover rate is only ∼1% and most cell lines still maintain a relatively low stable level, the mitochondrial carryover level of particular cell lines may rise sharply to 80% within several generations and then completely replace donor mitochondria in subsequent passages.^21^, ^24^ In mice, even if there are only 5% pathogenic mitochondria in the zygotic stage, it may reach 22% at birth, 30% before weaning, and 78% in their lifetime.^25^ If these cells differentiate into mitochondria‐dependent cells during embryonic development in vivo, it may seriously endanger the life of the new‐borns. Although there are many theories to explain this phenomenon, including nuclear–mitochondrial compatibility and mitochondrial competitiveness, there is no consensus to date.^19^, ^20^, ^26^, ^27^ Moreover, some studies have shown that gene editing or chemical methods can also modify or eliminate pathogenic mitochondria. However, the use of these technologies in clinical treatment may introduce biological and ethical risks.^28^, ^29^, ^30^, ^31^, ^32^ Therefore, the use of physically based methods to maximize the clearance of the mitochondrial carryover may be the only feasible scheme in future clinical treatment.

Among the main physically based MR schemes, polar body transfer (first polar body transfer [PB1T] and second polar body transfer [PB2T]) is considered to carry the fewest mitochondria.^11^, ^12^, ^13^ Especially in the PB2T scheme, the transfer operation is carried out after intracytoplasmic sperm injection (ICSI), so it reduces the oocyte disintegration rate by avoiding post‐replacement ICSI (in PB1T or SP transfer scheme). In addition, since the subsequent process of PB2T is the formation of female pronucleus, rather than the second meiosis without nuclear membrane protection, it will be more conducive to protecting the integrity and stability of genome.^11^, ^12^ However, the PB2T scheme (here, we call it the conventional PB2T scheme [cPB2T]) is carried out approximately 4 h after PB2 extrusion^11^, ^12^: with the increase in time after PB2 extrusion, the degree of DNA damage in the PB2 nucleus will increase, and the female pronucleus from PB2 cannot be fully unfolded due to the later PB2T, which may lead to a potential developmental crisis^33^; moreover, even though cPB2T is already the scheme with the fewest original mitochondria, the mitochondrial carryover can still be detected in the formed blastocysts and born offspring (∼1.7% in mice and ∼0.4% in humans).^11^, ^12^, ^13^

To solve the above problems, in this study, we established an artificial PB2 separation technology to achieve the transfer of PB2 within 0.5–1 h after PB2 extrusion. This will greatly avoid the DNA damage accumulation and female pronucleus unfolding disorder after PB2 extrusion (here, we call it early PB2T [ePB2T]). In addition, by the retained SP protrusion on PB2, we accurately positioned the location of PB2 integration into female pre‐pronucleus (FPPN)‐enucleated oocytes. Subsequently, we removed a small amount of cytoplasm around the fusion site to further reduce the mitochondrial carryover (here, we call it secondary residue removal [SRR]).

## 2 RESULTS

### 2.1 Optimization of the cPB2T scheme

**FIGURE 1:**
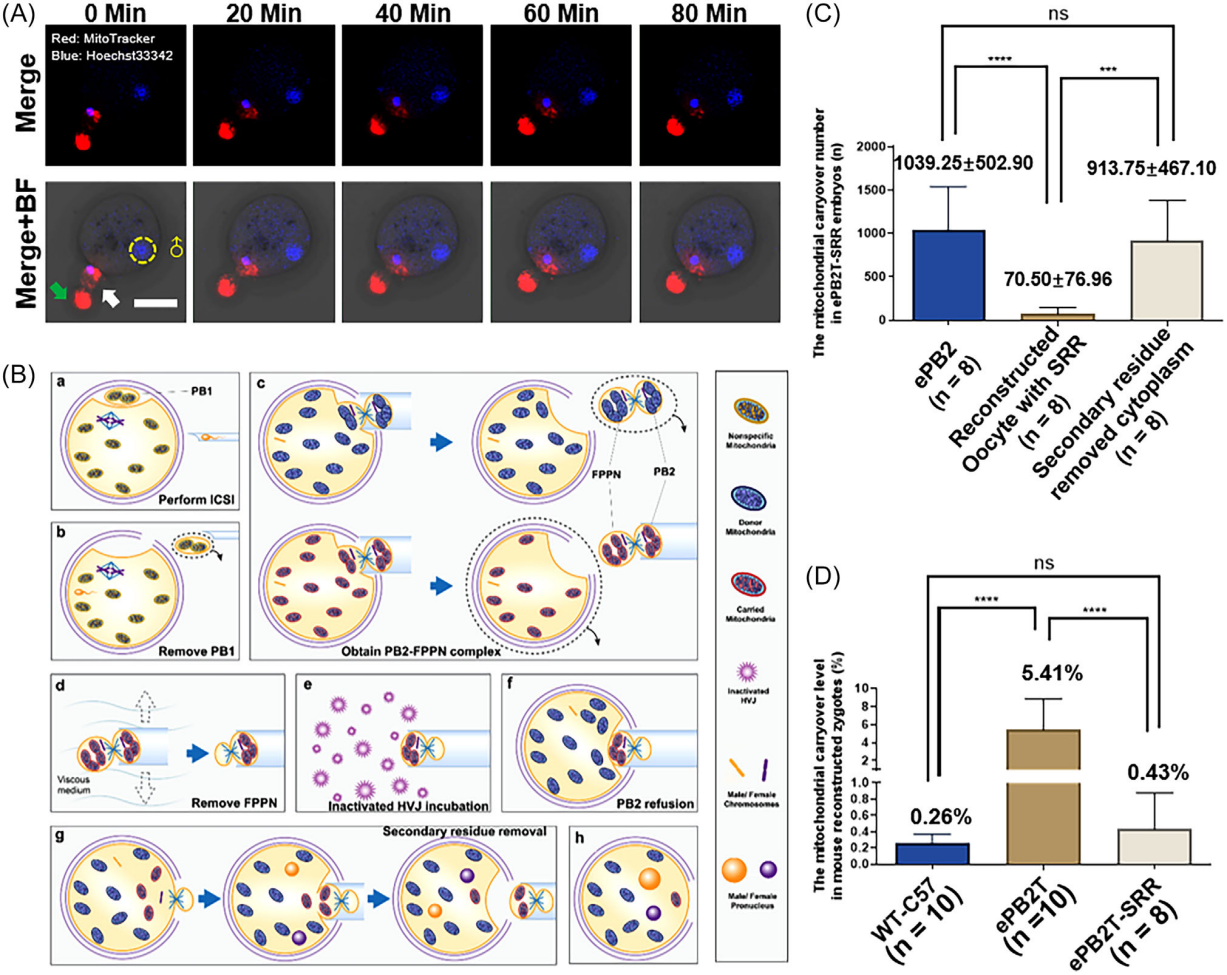
The optimized early second polar body transfer (ePB2T)‐secondary residue removal (SRR) scheme and its advantages in removing the original mitochondria. (A) Representative image of the male and female pronucleus and mitochondrial carryover migration in the mouse reconstructed zygotes (0/20/40/60/80 min post‐reconstruction), pronucleus (Hoechst33342, blue), and mitochondrial carryover (MitoTracker, red). The white arrow indicates the second polar body (PB2) body, the green arrow indicates the spindle (SP) protrusion, and the “♂” indicates the male pronucleus. Scale bars, 50 µm. (B) Process of ePB2T‐SRR scheme (a–h): (a) perform intracytoplasmic sperm injection (ICSI) on cytoplasmic and PB2 donor oocytes; (b) remove the first polar body (if necessary); (c) obtain the PB2–female pre‐pronucleus (FPPN) complex within 0.5 h of PB2 extrusion; (d) separate the FPPN from the PB2–SP complex in a modified viscous medium; (e) incubate the PB2 with inactivated Sendai virus; (f) oppress PB2 to the membrane of the enucleated cytoplasmic donor; (g) perform the second residue removal after 1–2 h of PB2 fusion; (h) perform the subsequent culture. (C) Quantification of the mouse mitochondrial carryover number in PB2 (*n* = 8), reconstructed oocytes of ePB2T‐SRR (*n* = 8) and secondary residue‐removed cytoplasm (*n* = 8) samples (*X*‐axis). The bar and whiskers represent the mean and standard deviation (SD). One‐way analysis of variance (ANOVA) demonstrated significant differences among the three groups, and the least significant difference (LSD) multiple comparisons test was performed to compare the significance among the groups, ^***^ *p* < 0.001, ^****^ *p* < 0.0001. (D) Quantification of the mouse reconstructed zygote mitochondrial carryover level (mitochondrial carryover number/total mitochondria number, %, *Y*‐axis) in the wild‐type (WT)‐C57 (*n* = 10), ePB2T (*n* = 10), and ePB2T‐SRR (*n* = 8) groups (*X*‐axis). The bar and whiskers represent the mean and SD. One‐way ANOVA demonstrated significant differences among the three groups, LSD multiple comparisons tests were performed to compare the significance among the groups, ^****^ *p* < 0.0001.

In the cPB2T scheme, a considerable proportion of PB2‐derived female pronucleus were significantly smaller than normal female pronucleus, and there was a significant increase in DNA damage (Figure S1A–C, cPB2T group). This impairs the developmental potential of the reconstructed embryo (Figure S2A–C). Moreover, through mitochondrial staining, we found that the mitochondrial carryover was carried into the FPPN‐enucleated oocytes. However, due to the traction of the ooplasm on the PB2 cytoplasm during fusion, we observed that for a period of time, the mitochondria were mainly concentrated near the fusion point, while the nucleus of PB2 was located deeper into the ooplasm (Figure 1A and Movie S1). In addition, we also observed that during the second meiosis of the mouse oocytes after fertilization, the SP connecting PB2 and the fertilized oocytes was still relatively robust in the early stage of PB2 extrusion, rather than dim and fragile after complete separation (Figure S3). This SP protruded continuously at the site of membrane fusion after PB2T (Figure S4). This suggests that if transfer is performed at an early stage of PB2 extrusion, the robust SP may become a marker of the PB2 integration site, which has residual mitochondria carried by the transferred PB2. Considering this hypothesis, we designed a scheme for transfer during the early stage of PB2 extrusion (ePB2T) and with further SRR (ePB2T‐SRR, Figure 1B and Movie S2).

To complete the operation of ePB2T as early as possible, we optimized the cPB2T program. After ICSI (Figure 1B‐a), we removed PB1 to avoid confusion with the subsequently extruded PB2 (Figure 1B‐b). Similar to methods described in the published research,^12^ we stained the nucleus of the FPPN–PB2 complex and observed that the FPPN is very close to the SP within 0.5–1 h after PB2 extrusion, suggesting that PB2 should occur together with the SP and FPPN at this time (Figures 1B‐c and S5). Then, the FPPN was artificially separated from the PB2–SP complex (the SP remained connected to the PB2) by gentle mechanical swinging in a modified viscous medium (Figure 1B‐d); without our swinging operation, approximately 4 h were needed for separation of the FPPN and PB2 in natural culture,^12^ which may have some risks to DNA integrity.^33^ Subsequently, we turned the PB2–SP direction and immersed part of the PB2 membrane in inactivated Sendai virus to obtain intercellular fusion ability (Figure 1B‐e). Then, the PB2 was compressed against FPPN‐enucleated oocytes to allow full contact between them, which promoted the fusion of the two parts (Figure 1B‐f). After fusion, we observed that the SP will still protrude at the fusion site. Therefore, we can remove the protruded SP and the adjacent ooplasm by performing the SRR step (carefully avoiding the initially apparent female pronucleus) to further reduce the mitochondrial carryover 1–2 h post‐PB2 fusion (Figure 1B‐g).

### 2.2 The ePB2T‐SRR scheme is conducive to mitochondrial carryover removal and protection of the main genetic material

To validate our scheme, we selected C57BL/6 oocytes as cytoplasmic donors and BALB/c oocytes as PB2 donors. After reconstruction and SRR, we first detected and compared the mitochondrial carryover levels between ePB2 and the reconstructed oocytes of ePB2T‐SRR and secondary residue‐removed cytoplasm (Figure 1C) by digital polymerase chain reaction (digital PCR, Table S1). As shown in the figure, most mitochondria carried by ePB2 (1039.25 ± 502.90, *n* = 8) were detected in the secondary residue‐removed cytoplasm (913.75 ± 467.10, *n* = 8). However, in the reconstructed oocyte after the SRR operation, the original carried mitochondrial level (70.50 ± 76.96, *n* = 8) decreased significantly. Then, we compared this level in the reconstructed oocytes between the ePB2T and ePB2T‐SRR groups (Figure 1D). Compared with the non‐reconstructed zygotes (C57BL/6) group (WT‐C57 group, 0.26 ± 0.11%, *n* = 10), the ePB2T group (5.41 ± 3.43%, *n* = 10) showed a much higher mitochondrial carryover residue level (WT‐C57 vs. ePB2T, *p* < 0.0001), which is very similar to the cPB2T scheme (Figure S2D). In the ePB2T‐SRR group (0.43 ± 0.44%, *n* = 8), we noticed that the residue level was significantly lower than that in the ePB2T group (ePB2T vs. ePB2T‐SRR, *p* = 0.0002), but there was no difference from the WT‐C57 group (WT‐C57 vs. ePB2T‐SRR, *p* = 0.86).

**FIGURE 2:**
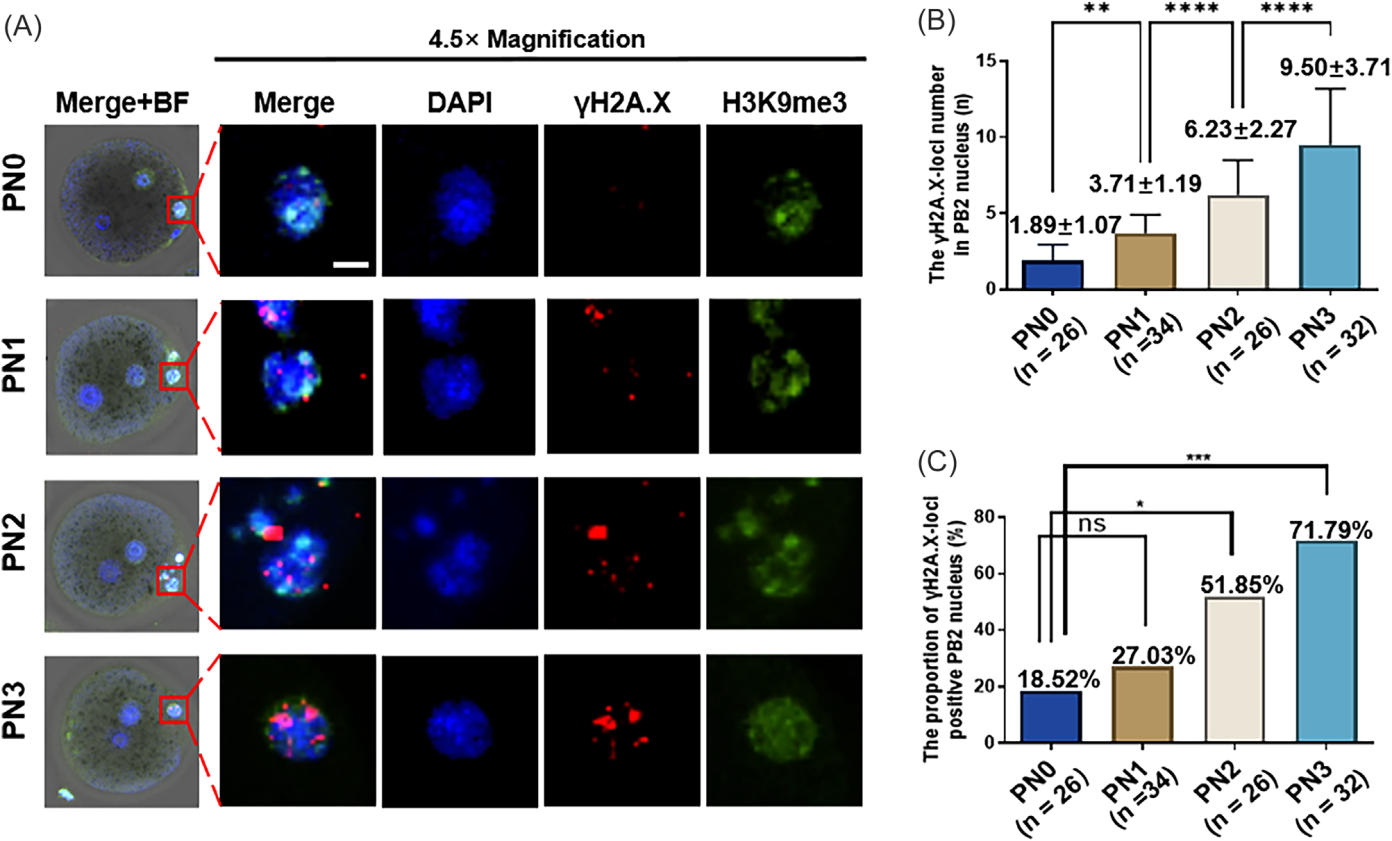
Trend of DNA damage in the second polar body (PB2) nucleus with time after mouse oocyte fertilization. (A) Representative images of DNA damage (γH2A.X based) in PB2 of zygotic stage embryos with different PB2 extrusion times in the PN0, PN1, PN2, and PN3 groups. γH2A.X (red), H3K9me3 (green, used to indicate the female pronucleus), and DAPI (blue). The red box indicates the PB2 nucleus, and there is a 4.5× magnification for PB2. Scale bars, 5 µm. (B) Quantification of the PB2 nucleus γH2A.X‐loci number (*Y*‐axis) in the PN0 (*n* = 26), PN1 (*n* = 34), PN2 (*n* = 26), and PN3 (*n* = 32) groups (*X*‐axis). The bar and whiskers represent the mean and standard deviation (SD). One‐way analysis of variance (ANOVA) demonstrated significant differences among the three groups. Least significant difference (LSD) multiple comparisons tests were performed to compare the significance among the groups, ^**^ *p* < 0.01, ^****^ *p* < 0.0001. (C) Quantification of the PB2 DNA damage rate (%, *Y*‐axis) in the PN0 (*n* = 26), PN1 (*n* = 34), PN2 (*n* = 26), and PN3 (*n* = 32) groups (*X*‐axis). Chi‐square test, ^*^ *p* < 0.05, ^***^ *p* < 0.001.

Moreover, in the cPB2T scheme, the time window for PB2T is relatively wide.^11^, ^12^ However, PB2 tends to undergo apoptosis and DNA damage due to its separation from the main cytoplasm.^33^ Therefore, we established an accurate correlation between the degree of DNA damage and the extrusion time of PB2 based on the comparison of γH2A.X‐loci number (Figure 2A–C). The results showed that compared with the PN0 stage zygote (PB2 extrusion for 0.5‐h PN0 group, 1.89 ± 1.07, *n* = 26), the γH2A.X‐loci number in the PB2 nucleus increased to 3.71 ± 1.19 (PB2 extrusion for the 1‐h PN1 group, *n* = 34; PN0 vs. PN1, *p* = 0.0036), 6.23 ± 2.27 (PB2 extrusion for the 2‐h PN2 group, *n* = 26; PN1 vs. PN2, *p* < 0.0001), and 9.50 ± 3.71 (PB2 extrusion for the 4‐h PN3 group, *n* = 32; PN2 vs. PN3, *p* < 0.0001) (Figure 2B). Taking the significant γH2A.X loci in the PB2 nucleus as the positive index, we also found that the positive proportion increased with the extension of PB2 extrusion time (PN0 group: 18.52%, *n* = 26; PN1 group: 27.03%, *n* = 34; PN2 group: 51.85%, *n* = 26; PN3 group: 71.79%, *n* = 32; Figure 2C). This suggests that PB2T should be carried out as soon as possible to avoid the potential adverse effects of DNA damage on the offspring. Therefore, in our designed ePB2T and ePB2T‐SRR, the time from PB2 extrusion to PB2 refusion was controlled within approximately 1 h as much as possible. In addition, we also conducted DNA damage identification on the reconstructed zygote at late PN stage, and the results showed that our operation would make female pronucleus normal in size and did not introduce new DNA damage (Figure S1A–C, ePB2T and ePB2T‐SRR groups).

### 2.3 Efficiency of the ePB2T‐SRR scheme and preimplantation development of reconstructed embryos in mice

To further confirm whether the ePB2T‐SRR scheme is feasible in practical applications, we first evaluated the success rate of each step of this scheme. After performing ICSI to obtain fertilized oocytes,^34^ we performed reconstruction operations (including removing the PB2–FPPN complex from fertilized oocytes, obtaining PB2 with SP, incubating with Sendai virus and fusing again), and we noticed that the efficiency of successful refusion was 84.07% (*n* = 113). Then, we divided the reconstructed fertilized oocytes into two groups: one was directly cultured (ePB2T group, *n* = 36) and the other group of oocytes underwent SRR operation (ePB2T‐SRR group, *n* = 59), and the success rate of this SRR operation was 83.05%.

**FIGURE 3:**
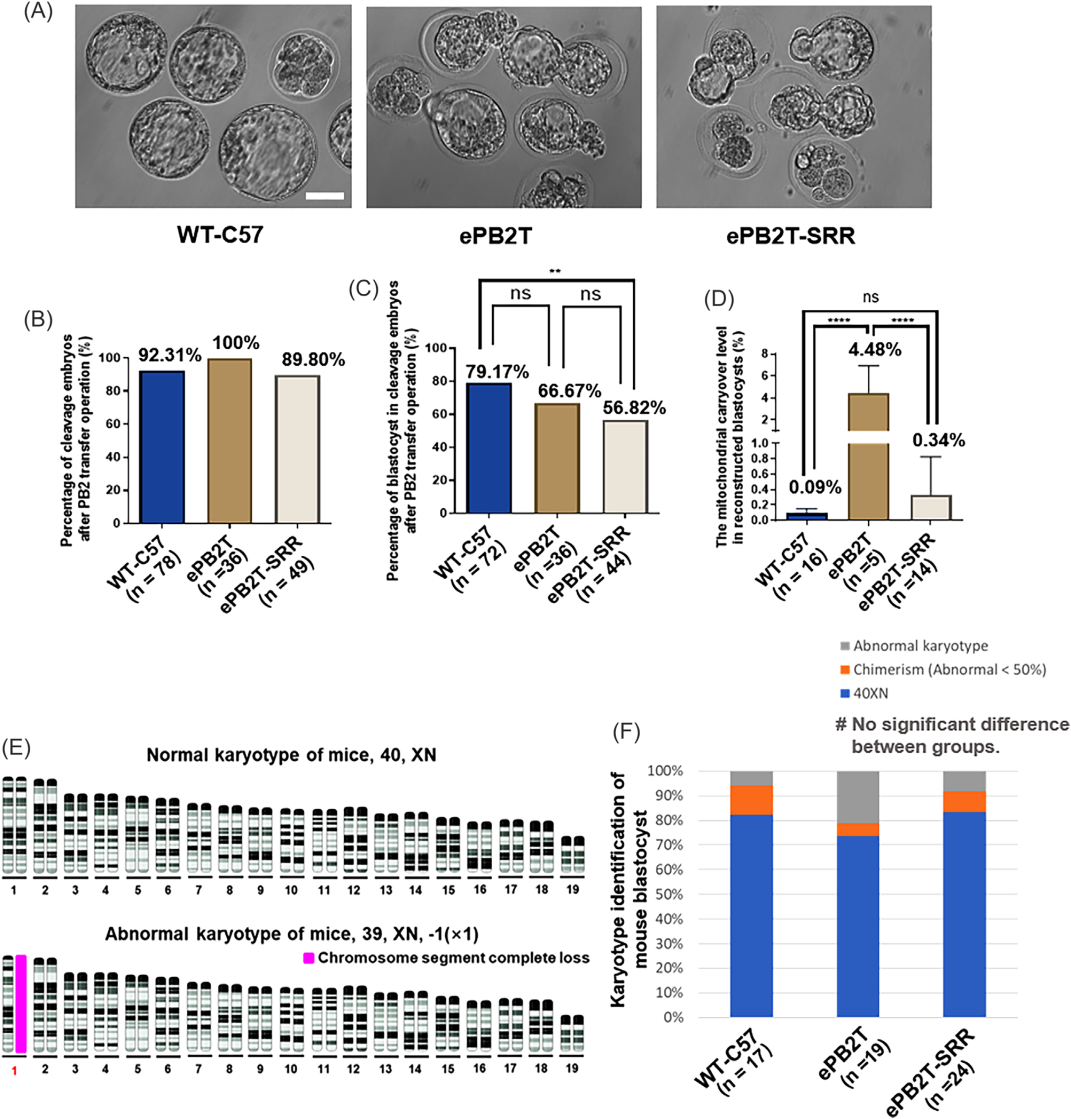
Efficiency of the early second polar body transfer (ePB2T)‐secondary residue removal (SRR) scheme and genomic stability of the reconstructed embryos. (A) Representative images of blastocyst formation in the wild‐type (WT)‐C57, ePB2T, and ePB2T‐SRR groups. Scale bars, 50 µm. (B) Quantification of the embryo cleavage rate (%, *Y*‐axis) in the WT‐C57 (*n* = 78), ePB2T (*n* = 36), and ePB2T‐SRR (*n* = 49) groups (*X*‐axis). Chi‐square test, *p* > 0.05. (C) Quantification of the blastocyst formation rate (%, *Y*‐axis) in the WT‐C57 (*n* = 72), ePB2T (*n* = 36), and ePB2T‐SRR (*n* = 44) groups (*X*‐axis). Chi‐square test, ^**^ *p* < 0.01. (D) Quantification of the mouse reconstructed blastocyst mitochondrial carryover level (mitochondrial carryover number/total mitochondria number, %, *Y*‐axis) in the WT‐C57 (*n* = 16), ePB2T (*n* = 5), and ePB2T‐SRR (*n* = 14) groups (*X*‐axis). The bar and whiskers represent the mean and standard deviation (SD). One‐way analysis of variance (ANOVA) demonstrated significant differences among the three groups, and least significant difference (LSD) multiple comparisons tests were performed to compare the significance among the groups, ^****^ *p* < 0.0001. (E) Representative images of normal and abnormal mouse blastocyst karyotypes (the X and Y chromosomes are not shown). The number represents the sequence number of the chromosome pair, and the red number indicates abnormal chromosome number, and the solid pink square represents chromosome segment complete loss. (F) Quantification of blastocysts with normal karyotypes (%, *Y*‐axis) in the WT‐C57 (*n* = 17), ePB2T (*n* = 19), and ePB2T‐SRR (*n* = 24) groups (*X*‐axis). Chi‐square test, *p* > 0.05.

We then evaluated the preimplantation embryo development of the two groups (Figure 3A–C). Compared with the WT‐C57 group (92.31%, *n* = 78) fertilized based on ICSI and without replacement operation, neither the ePB2T group (100%, *n* = 36; WT‐C57 vs. ePB2T, *p* = 0.0873) nor the ePB2T‐SRR group (89.80%, *n* = 49; WT‐C57 vs. ePB2T‐SRR, *p* = 0.6242) showed a significant difference in cleavage rate (Figure 3B). In terms of the blastocyst formation rate (Figure 3C), we noticed that the rates of both the ePB2T (66.67%, *n* = 36) and ePB2T‐SRR (56.82%, *n* = 44) groups were higher than that of the cPB2T scheme (44.12%, *n* = 34, Figure S2C), although the ePB2T‐SRR group's rate was significantly lower than that of the WT‐C57 group (79.17%, *n* = 72; WT‐C57 vs. ePB2T‐SRR, *p* = 0.0103). These results suggest that our early transfer strategy is conducive to the development of reconstructed embryos, and the impact of the SRR strategy on embryonic development needs to be further evaluated. Considering that our operation may affect the stability of chromosomes, we identified the karyotype of the blastocysts by preimplantation genetic testing for structural chromosomal rearrangements (PGT‐A technology) (Figure 3E,F). The results showed that the proportion of normal karyotypes (including low‐proportion chimerism, Figure 3F and Table S2) was 94.12% (*n* = 17) in the WT‐C57 group, 78.95% in the ePB2T group (*n* = 19), and 91.67% in the ePB2T‐SRR group (*n* = 24).

We also detected the mitochondrial carryover level of the blastocysts in each group (Figure 3D). Compared with the background value of the WT‐C57 group (0.09 ± 0.06%, *n* = 16), the content of the mitochondrial carryover in the ePB2T group (4.48 ± 2.44%, *n* = 5; WT‐C57 vs. ePB2T, *p <* 0.0001) was significantly higher. However, the ePB2T‐SRR group (0.34 ± 0.49%, *n* = 14; WT‐C57 vs. ePB2T‐SRR, *p* = 0.4765) did not show a significant difference from the WT‐C57 groups. This suggests that our secondary removal of residues can effectively reduce the original mitochondria, and our operation has little impact on the stability of the genome.

### 2.4 Development and genetic drift analysis of reconstructed embryos in mice

**FIGURE 4:**
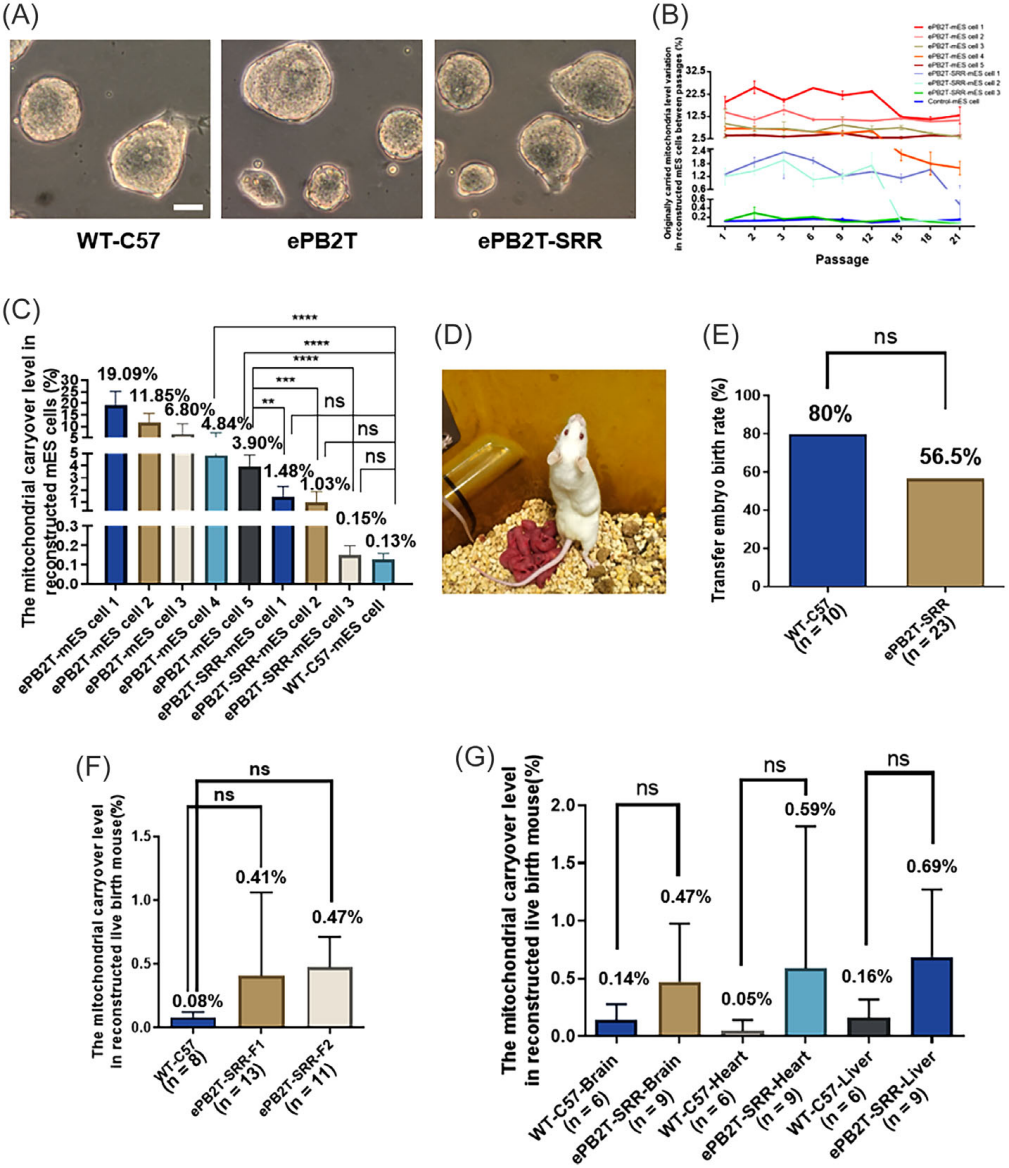
Developmental potential and genetic drift of the reconstructed embryos. (A) Representative images of the derived mouse embryonic stem (mES) cells in the wild‐type (WT)‐C57, early second polar body transfer (ePB2T) and ePB2T‐secondary residue removal (SRR) groups. Scale bars, 50 µm. (B) Heteroplasmic mitochondrial DNA (mtDNA) carryover (%, *Y*‐axis) in the mES cell line during passages (*X*‐axis) of the WT‐C57, ePB2T, and ePB2T‐SRR groups. (C) Means of mtDNA carryover in each mES cell line. The bar and whiskers represent the mean and standard deviation (SD). One‐way analysis of variance (ANOVA) demonstrated significant differences among the groups, and least significant difference (LSD) multiple comparisons tests were performed to compare the significance among the groups, ^**^ *p* < 0.01, ^***^ *p* < 0.001, ^****^ *p* < 0.0001. (D) Representative image of the reconstructed embryonic mice born to the ePB2T‐SRR group. (E) Quantification of the reconstructed embryonic mouse birth rate (%, *Y*‐axis) in the WT‐C57 (*n* = 10) and ePB2T‐SRR (*n* = 23) groups (*X*‐axis). Chi‐square test, *p* > 0.05. (F) Quantification of the mitochondrial carryover level (mitochondrial carryover number/total mitochondria number, %, *Y*‐axis) in the WT‐C57 (*n* = 8), ePB2T‐SRR‐F1 (*n* = 13), and ePB2T‐SRR‐F2 (*n* = 11) groups (*X*‐axis). The bar and whiskers represent the mean and SD. One‐way ANOVA demonstrated significant differences among the three groups, LSD multiple comparisons tests were performed to compare the significance among the groups, *p* > 0.05. (G) Quantification of the F1 reconstructed mouse mitochondrial carryover level (mitochondrial carryover number/total mitochondria number, %, *Y*‐axis) in different organs. WT‐C57‐Brain (*n* = 6) versus ePB2T‐SRR‐Brain (*n* = 9), WT‐C57‐Heart (*n* = 6) versus ePB2T‐SRR‐Heart (*n* = 9), and WT‐C57‐Liver (*n* = 6) versus ePB2T‐SRR‐Liver (*n* = 9) (*X*‐axis). The bar and whiskers represent the mean and SD. The bar and whiskers represent the mean and SD. *t*‐Test were performed to compare significance between groups, *p* > 0.05.

To evaluate the developmental potential and mitochondrial drift of reconstructed mouse embryos, we derived five ePB2T and three ePB2T‐SRR mouse embryonic stem (mES) cell lines from reconstructed embryos (Figure 4A) and then detected the mitochondrial carryover level of their passaged cells (Figure 4B,C). Our data show that mtDNA carryover of the ePB2T‐mES cells and ePB2T‐SSR‐mES cells (Figure 4B) fluctuated within a relatively stable range from P1 to P21. Compared with the ePB2T‐mES cell lines, ePB2‐SSR‐mES cells show a smaller fluctuation range. More importantly, we observed that when the mitochondrial carryover in the primary cells (P1) was below a certain level, it seemed to decrease even further with cell passage (ePB2T‐mES cell 4, ePB2T‐SRR‐mES cell 1, ePB2T‐SRR‐mES cell 2). In addition, the average contents of the mitochondrial carryover in ePB2T‐ES cells were 19.09 ± 6.12% (ePB2T‐mES cell 1, *n* = 27), 11.85 ± 3.81% (ePB2T‐mES cell 2, *n* = 27), 6.80 ± 4.44% (ePB2T‐mES cell 3, *n* = 26), 4.84 ± 2.50% (ePB2T‐mES cell 4, *n* = 26), and 3.90 ± 0.96% (ePB2T‐mES cell 5, *n* = 27), and in ePB2T‐SSR‐ES cells, they were 1.48 ± 0.83% (ePB2T‐SRR‐mES cell 1, *n* = 27), 1.03 ± 0.87% (ePB2T‐SRR‐mES cell 2, *n* = 26), and 0.15 ± 0.05% (ePB2T‐SRR‐mES cell 3, *n* = 26) (Figure 4C). Importantly, there was no difference in heterogeneity between the WT‐C57 mES cells (0.13 ± 0.03%, *n* = 27) and the ePB2T‐SRR‐mES cells 1/2/3 (based on one‐way analysis of variance [ANOVA] and least significant difference [LSD] multiple comparisons tests; ePB2T‐SRR‐mES cell 1 vs. WT‐C57‐mES cell, *p* = 0.0994; ePB2T‐SRR‐mES cell 2 vs. WT‐C57‐mES cell, *p* = 0.2724; ePB2T‐SRR‐mES cell 3 vs. WT‐C57‐mES cell, *p* = 0.9777). In addition, our data demonstrated that ePB2T‐mES cells with higher mitochondrial carryover levels exhibit a higher standard deviation (SD) value (Figure S6) than ePB2T‐SSR‐mES cells, suggesting that mES cells with high mitochondrial carryover levels have a higher risk of heterogenetic mtDNA drift.

Then, we performed the surrogate transfer of mouse embryos reconstructed with the ePB2T‐SRR scheme and obtained born mice of F1 generation (Figure 4D,E). Compared with the simultaneous surrogacy transfer WT‐C57 group (80%, *n* = 10), the birth rate of the ePB2T‐SRR group (56.5%, *n* = 23) was relatively low but without a significant difference. Then, we obtained F2 generation through F1 inbreeding. Next, we also detected the mitochondrial carryover level of the new‐born mice (Figure 4F); compared with the WT‐C57 group (0.08 ± 0.04%, *n* = 8), both the ePB2T‐SRR F1 group (0.41 ± 0.65%, *n* = 13; WT‐C57 vs. ePB2T‐SRR‐F1, *p* = 0.1134) and the ePB2T‐SRR F2 group (0.47 ± 0.24%, *n* = 11; WT‐C57 vs. ePB2T‐SRR‐F2, *p* = 0.0666) showed only a slight increase (no significance) in mitochondrial residue, which was lower than the level of the cPB2T scheme (1.7%) reported in the literature.^11^

Subsequently, we compared the adult weights (8 weeks old) of WT‐C57 and reconstructed F1/F2 mice (Figure S7). The results showed that there was no difference in adult weight between the WT‐C57 group, the F1 reconstructed mouse group and the F2 reconstructed mouse group. Karyotype identification showed that the chromosomes of these adult mice were normal, and their inbreeding could also produce normal F2 generation mice (Table S3). Then, we sacrificed a portion of the F1 male mice and detected the mitochondrial carryover residual level in their important organs that depend on mitochondrial function (brain, heart, and liver, Figure 4G). The results showed that compared with the wild‐type (WT)‐C57 group, there was no significant difference in mitochondrial residue in these organs (WT‐C57 vs. F1 group: 0.14 ± 0.14%, *n* = 6 vs. 0.47 ± 0.50%, *n* = 9 in brain, *p* = 0.1410; 0.05 ± 0.09%, *n* = 6 vs. 0.59 ± 1.23%, *n* = 9 in heart, *p* = 0.3060; 0.16 ± 0.16%, *n* = 6 vs. 0.69 ± 0.58%, *n* = 9 in liver, *p* = 0.0513).

Combining the research in this part, we believe that the reconstructed embryo based on our ePB2T‐SRR scheme has complete developmental potential. Even after the lowest content of MR (PB2T), reliable SRR is still needed. Our ePB2T‐SRR scheme provides a feasible choice for SRR.

### 2.5 Validating the ePB2T‐SRR scheme in humans

**FIGURE 5:**
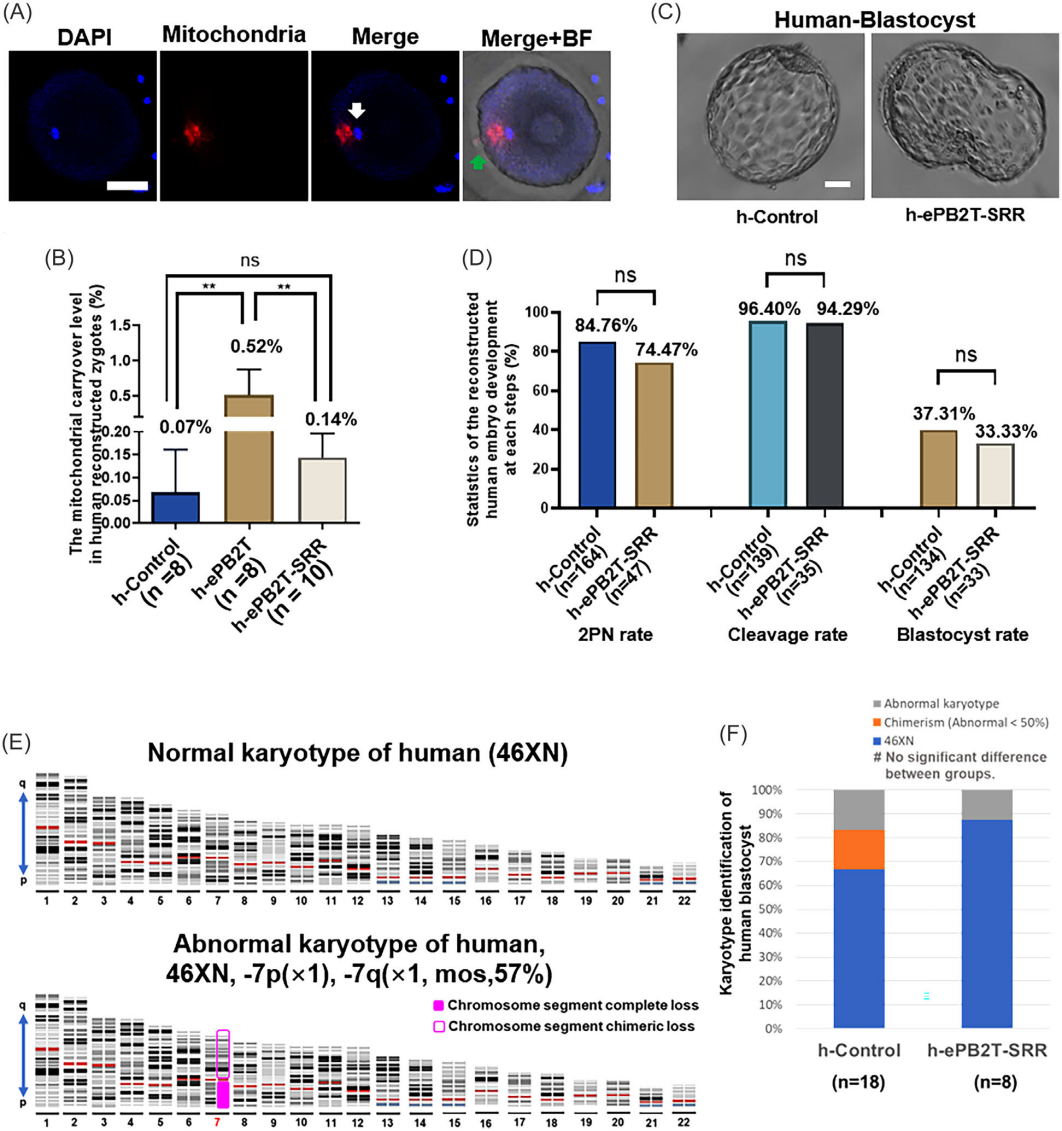
Validation of the early second polar body transfer (ePB2T)‐secondary residue removal (SRR) scheme in human embryos. (A) Representative image of the second polar body (PB2) nucleus and mitochondrial carryover location in the human reconstructed zygotes, DAPI (blue), mitochondrial carryover (red). The white arrow indicates the PB2 nucleus and the green arrow indicates the spindle (SP) protrusion. Scale bars, 40 µm. (B) Quantification of the human reconstructed zygote mitochondrial carryover level (mitochondrial carryover number/total mitochondria number, %, *Y*‐axis) in the h‐Control (*n* = 8), h‐ePB2T (*n* = 5), and h‐ePB2T‐SRR (*n* = 6) groups (*X*‐axis). The bar and whiskers represent the mean and standard deviation (SD). One‐way analysis of variance (ANOVA) demonstrated significant differences among the three groups, and least significant difference (LSD) multiple comparisons tests were performed to compare the significance between the groups, ^**^ *p* < 0.01. (C) Representative images of the reconstructed human blastocyst in the h‐Control and h‐ePB2T‐SRR groups. Scale bars, 50 µm. (D) The normal development rate (%, *Y*‐axis) of the h‐Control and h‐ePB2T‐SRR embryos at the steps of 2PN pronucleus (h‐Control, *n* = 164; h‐ePB2T‐SRR, *n* = 47), cleavage (h‐Control, *n* = 139; h‐ePB2T‐SRR, *n* = 35), and blastocyst formation (h‐Control, *n* = 134; h‐ePB2T‐SRR, *n* = 33) (*X*‐axis). (E) Representative images of normal and abnormal human blastocyst karyotypes (the X and Y chromosomes are not shown). The *q* direction is the long arm of chromosome and the *p* direction is the short arm of chromosome. The number represents the sequence number of the chromosome pair, the red number indicates abnormal chromosome number, the solid pink square represents chromosome segment complete loss, and the hollow pink square represents the chromosome segment chimeric loss. (F) Quantification of the human reconstructed blastocyst karyotype (%, *Y*‐axis) in the h‐Control (*n* = 18) and h‐ePB2T‐SRR (*n* = 8) groups, Chi‐square test, *p* > 0.05.

After evaluating the ePB2T‐SRR scheme in a mouse model, we wanted to know whether this scheme is feasible in humans. Therefore, we used oocytes donated by research volunteers to verify our scheme (Figure 5 and Movie S3). First, we observed that whether human oocyte has similar characteristics to mouse oocyte after ePB2T scheme replacement. The results showed that after the PB2–SP complex was integrated into human oocytes, the mitochondrial carryover also showed a tendency to separate from the nucleus of PB2 (white arrow), and the SP (green arrow) also protruded at the fusion site (Figure 5A). In addition, we also noted that there was no significant ooplasm loss in zygotes after the ePB2T or ePB2T‐SRR operation (Figure S8). Then, based on the naturally neutral deletion (mtDNA.8271‐8279del) of human mtDNA,^35^, ^36^ we performed an ePB2T‐SRR operation between WT and mtDNA.8271‐8279del fertilized human oocytes and detected the efficiency of SRR by digital PCR analysis (Figure 5B and Table S1). The results showed that the secondary residual removal operation was also meaningful in human PB2T. Compared with the human ePB2T group (h‐ePB2T, 0.52 ± 0.36%, *n* = 8) without SRR, the mitochondrial carryover level in the human ePB2T‐SRR group (h‐ePB2T‐SRR, 0.14 ± 0.05%, *n* = 10; h‐ePB2T vs. h‐ePB2T‐SRR, *p* = 0.0011) decreased significantly, although there was no difference from the background value of the human control group (h‐Control, 0.07 ± 0.09%, *n* = 8; h‐Control vs. h‐ePB2T‐SRR, *p* = 0.4490). Subsequently, we performed statistical analysis of the development of human ePB2T‐SRR reconstructed embryos (Figure 5C,D). Based on fertilized oocytes with PB2 extrusion, after our operation, 74.47% of zygotes (*n* = 47) exhibited 2PN. The cleavage rate of the 2PN zygotes was 94.29% (*n* = 35). The proportion of blastocysts formed from the cleaved embryos was 33.33% (*n* = 33). This is close to the clinical outcome of oocyte donors (84.76% for 2PN rate, *n* = 164; 96.40% for cleavage rate, *n* = 139; 37.31% for blastocyst rate, *n* = 134). Then, some of the blastocysts were identified by PGT‐A technology to determine their karyotypes (Figure 5E,F and Table S4). The results showed that 87.5% of the karyotypes in the h‐ePB2T‐SRR group (*n* = 8) were normal, while 83.3% of the samples in the h‐Control group (*n* = 18) were normal (including normal karyotypes with low‐level abnormal chimerism, abnormal rate <40%). These results suggest that after the SRR operation, the level of mitochondria in human reconstructed oocytes is further decreased and that these operations do not affect the integrity of human embryonic chromosomes. This indicates that the ePB2T‐SRR scheme would be conducive to the implementation in clinical treatment in the future.

## 3 DISCUSSION

Mitochondrial diseases bring great physical and mental pain, as well as economic pressure to patients and their families, and some of them may be fatal. The core strategy of treatment is to replace the mitochondria with pathogenic genes as completely as possible with normal mitochondria.^9^, ^12^, ^13^, ^14^, ^26^, ^37^ Earlier studies injected healthy mitochondria into human oocytes with pathogenic mitochondria and obtained live births.^37^ However, due to the low proportion of healthy mitochondria, the improvement of the offspring's quality of life is extremely limited. Moreover, biological (e.g., gene editing) or chemical (e.g., uncoupling agents) means are difficult to apply in clinical treatment in the short term because of their great uncertainty.^28^, ^29^, ^30^, ^31^, ^38^ Therefore, transferring the main genetic material into healthy enucleated oocytes is the most feasible scheme at present. However, previous reports and our current study have shown that even we choose the scheme of polar body transfer, which carries the smallest number of mitochondria, still cannot completely eliminate the mitochondrial carryover^11^, ^12^, ^13^: when the residual reaches a certain level, it greatly increases the possibility of pathogenic mitochondria drifting to a higher level.^21^, ^24^, ^25^ Therefore, it is necessary to develop a reliable technique for SRR to reduce the mitochondrial carryover level as much as possible.

In this study, we designed a scheme for earlier PB2T and used the protruded SP to perform SRR after PB2 and FPPN‐enucleated oocyte fusion. It could further remove the residue of original mitochondria. In addition, we also proved that the PB2 DNA damage accumulates with the extension of time after the extrusion of the PB2.^33^ Therefore, ePB2T is also conducive to protecting its nuclear genetic material from DNA damage caused by apoptosis and other factors. Then, we first validated this scheme in mouse oocytes and found that in the reconstructed zygotes, the level of mitochondrial carryover residue in the group with SRR was much lower than that in the group without SRR. Subsequent embryo culture experiments proved that a considerable proportion of reconstructed embryos (both ePB2T and ePB2T‐SRR groups) developed into blastocysts, significantly higher than the rate in cPB2T scheme. In addition, karyotype identification also showed that most of the blastocyst karyotypes of these two groups were normal. Most interestingly, in mice, we found that the normal‐karyotype rate of the ePB2T‐SRR group was higher than that of the ePB2T group. In our research, considering the relatively low blastocyst formation rate of the ePB2T‐SRR group (not significant), the proportion of blastocysts with normal karyotypes actually obtained in the ePB2T‐SRR and ePB2T groups was similar (taking the cleaved embryos of this group as the denominator, Figure S9). These results preliminarily show that our strategy of ePB2T is likely to avoid the potential PB2 nucleus DNA damage from apoptosis and is beneficial to the reconstructed embryo development. And the impact of our SRR operation on embryonic development is limited.

To further explore the impact of our operation on developmental potentiality and mitochondrial drift in reconstructed embryos, we performed mES cell derivation and surrogate transfer for reconstructed embryos and successfully obtained ES cell lines and livebirth mice. In the mitochondrial detection of each ES cell line, we found that the cell lines with lower initial mitochondrial carryover content had less variation between generations and within generations. And the detection in F1/F2 mice showed that the mitochondrial carryover level of the ePB2T‐SRR group was further reduced compared with that of the mice based on the PB2T scheme reported in the literature^11^ and was very close to that of our WT‐C57 group. And the detection in different organs of F1 generation mice also showed that there was no obvious difference in residual levels between different tissues. Through this part of research, we speculate that the distribution of mutant mitochondria in daughter cells is random during cell division. Therefore, when the level of initial mutant mitochondria is low to a certain extent, even if all of the mutant mitochondria are allocated to one of the daughter cells, they still cannot accumulate enough numbers to cause mitochondrial genetic drift. In addition, the F1/F2 body weight assessment, F2 generation from F1 inbreeding and karyotype identification of the F1/F2 generation also proved that the mice are healthy and able to maintain their lineage.

On the basis of the mouse research, we preliminarily verified our scheme in donor oocytes of human volunteers. The results show that our operation can not only effectively eliminate the residue of mitochondrial carryover but also maintain the reconstructed embryonic development at an acceptable level. Karyotype identification also showed that most of these blastocysts were normal embryos. Moreover, the proportion of normal karyotypes of reconstructed human embryos was even higher than that of the clinical control group. This may be because most embryos unsuitable for development were eliminated before blastocyst formation, and the sperm we used in this research were high‐quality sperm from healthy male volunteers. This means that it is preliminarily feasible to carry out this scheme in future clinical treatment. Our results suggest that the migration of mitochondria in the cytoplasm is not very rapid in human oocytes, so we can take secondary measures to remove residues. It also suggests that our operation causes minimal damage to the just‐separated human chromosomes in PB2. However, due to the limitation of the human oocyte number and ethics, we only evaluated the preimplantation development of human reconstructed embryos, but did not perform stem cell derivation and embryo transfer assessment. This means that more preclinical and clinical evaluations are needed for the future promotion and application of this technique.

In conclusion, based on the characteristics of the second meiosis after fertilization of oocytes, we designed an artificial PB2 separation technique to obtain PB2 with SP protrusion within 0.5–1 h after extrusion. This will enable MR based on the PB2T to be carried out earlier to reduce potential DNA damage and developmental crisis. Since the retained SP protrusion can mark the fusion site after PB2 fusion, it makes accurate SRR possible. In mice, we proved that this scheme could eliminate the mitochondrial carryover and also provide sufficient high‐quality embryos that can develop into healthy mice. In humans, we also proved the feasibility of this scheme to eliminate mitochondria and obtained a considerable proportion of high‐quality blastocysts. Although some preclinical and clinical work may be required for further validation, our results in this study still suggest that this technique may be the most valuable scheme for clinical MR therapy in the future.

## 4 METHODS AND MATERIALS

### 4.1 Research object and content and overall experimental design

The main experimental subjects of this study are mouse and human oocytes (and embryos developed from fertilized oocytes). The main research contents are the mitochondrial residual level and developmental potential of oocytes undergoing MR by ePB2T and ePB2T‐SRR schemes. Oocytes without MR were used as control group.

Oocyte samples were randomly placed in the culture medium before grouping. For experiments in which different groups cannot be operated or tested synchronously, the samples in the same group are divided into several subgroups, and the operation or detection is carried out alternately between subgroups in different groups.

The recorder of the experiment did not know the sample grouping information in advance. After obtaining the experimental results, a special person was assigned to carry out statistical analysis.

### 4.2 Oocyte and sperm collection

Mouse metaphase II (MII) oocytes were collected from WT C57BL/6 and BALB/c female mice (∼3–4 weeks old, Vitalriver, China) that had been induced to ovulate by the intraperitoneal injection of 10 IU of pregnant mare serum gonadotrophin (Ningbo No. 2 Hormone Factory, China) and 10 IU human chorionic gonadotrophin (hCG, Ningbo No. 2 Hormone Factory) at a 48‐h interval. Oocytes were recovered 14–16 h post‐hCG administration in modified HTF media (modified Human Tubal Fluid [mHTF], 90126, Irvine Scientific, USA). Mouse sperm samples were collected from the cauda epididymis and vas deferens of WT CD‐1/ICR male mice (∼10–12 weeks old, Vitalriver, China), and a 30‐min swim‐up at 37°C in mHTF medium was used to gather motile sperm.

For human oocyte collection, controlled ovarian stimulation was performed for patients undergoing In vitro fertilization and embryo transfer (IVF‐ET) treatment according to an individualized protocol. When at least one dominant follicle reached 20 mm in diameter or three dominant follicles reached 18 mm in diameter, ovulation was triggered by hCG 2000 IU (Lizhu Pharmaceutical Trading Co.) in conjunction with triptorelin 0.1 mg (Decapeptyl; Ferring Pharmaceuticals), followed by Transvaginal sonography (TVS)‐guided oocyte retrieval approximately 36 h later. All follicles with diameters greater than 10 mm were retrieved. Human sperm were collected from masturbation ejaculation and purified by gradient centrifugation to gather motile sperm.

The evaluation of gametes and embryos is based on the Cummins's criteria, and only the samples that meet the standard will be subject to subsequent experimental operations.

### 4.3 Oocyte processing, ICSI, and embryo culture

Cumulus oocyte complex (COC) was added to preheated mHTF (containing 5 µg/mL hyaluronidase/H4272, Sigma, Germany). After 20–30 s of incubation, these COCs were quickly transferred to fresh mHTF and repeatedly blown and aspirated with a slim pipette to remove the cumulus mass. Then, the cumulus‐free oocytes were rinsed in fresh mHTF three times. Subsequently, mature MII oocytes were selected and placed in the culture medium for 0.5–1 h to prepare for ICSI. For mice, optimized ICSI was performed as previously described.^34^ For humans, conventional ICSI was used for insemination. Fertilized mouse oocytes were placed in KSOM (MR‐107, Millipore, Germany) and maintained at 37°C and 5% CO_2_. Fertilized human oocytes were placed in G1 PLUS medium (10128, Vitrolife, Sweden) and maintained at 37°C and 6.0% CO_2_. On day 3 after fertilization, normally fertilized and cleaved embryos were transferred to G2 PLUS medium (10132, Vitrolife, Sweden) for subsequent culture until day 6 after fertilization. Scoring of gametes and embryos.

### 4.4 Mitochondria staining

Oocytes and PB2–FPPN complexes were transferred to the operation solution (mHTF with 2.5 µM cytochalasin B/CB, 14930‐96‐2, Sigma) containing MitoTracker (1:2000, M22426, Thermo, USA), stained for 20 min, and Hoechst33342 (E607302‐0005, Sangon) was used to mark the nucleus of living cells (37°C, 20 min). Then, the samples were transferred to fresh operation solution for 10 min to wash off any unbound dye. After the PB2T operation, the samples were imaged with an Olympus confocal microscope.

### 4.5 Oocyte and embryo immunostaining

The mouse embryos were fixed in 1% paraformaldehyde in phosphate‐buffered saline (PBS) at 4°C overnight. Then, the embryos were permeabilized with 0.25% Triton X‐100 at room temperature and blocked with 3% bovine serum albumin in PBS at 4°C overnight. Primary antibody incubation was conducted at 4°C overnight. The primary antibodies included polyclonal rabbit anti‐γH2A.X (ab11174, Abcam, USA) and monoclonal mouse anti‐H3K9me3 (MABI0308, GeneTex, USA). Then, the samples were incubated with DyLight 488/549/633‐conjugated goat anti‐mouse or rabbit immunoglobulin G (H + L) antibodies (#35502, #35557, or #35512, Thermo) at 4°C overnight, and the nuclei were stained with 4',6‐diamidino‐2‐phenylindole (DAPI). After staining, the samples were mounted with coverslips and imaged with an Olympus confocal microscope.^39^

### 4.6 Performing the ePB2T‐SRR operation

The details are presented in Figure 1B, Table S5, and Movies S2 and S3. After ICSI, the zonal pellucida was perforated by microlaser (ensuring that the SP or PB2 would not be damaged, 150 ms, ZILOS‐tk, Hamilton Thorne, USA). Then, the oocytes of mice and humans with the PB1 removed (most mouse first polar bodies are naturally apoptotic) were put into the time‐lapse device to observe the extrusion of the PB2. Once the PB2 was extruded, these oocytes were transferred to mHTF containing 2.5 µM cytochalasin B (CB, 14930‐96‐2, Sigma) for 5–10 min. Then, under SP observation microscope, PB2 and a certain volume (∼1.5× volume of PB2 for mouse, ∼2× volume of PB2 for human) of cytoplasm (containing FPPNs) connected to the PB2 through the SP (PB2–FPPN complex) were carefully removed from the oocytes by a 15 µm flat biopsy needle. After carefully shaking off the FPPN, the direction of the PB2–SP was turned to make half of the cytoplasm of the PB2 come into contact with inactivated Sendai virus (40 s for mouse, 60 s for human). It was then transferred to an oocyte with the PB2–FPPN complex removed, and the PB2–SP was gently pressed to make full contact with the oocyte. The PB2 will fuse with the oocyte within half an hour, but the SP will remain at the fusion site. Approximately 1 h after fusion, a 12 µm flat biopsy needle was used to suck out cytoplasm (0.5–1× PB2 volume for mouse, ∼1× PB2 volume for human) with the SP as the fusion site marker, and the removed cytoplasm was carefully separated from the oocyte. Then, these reconstructed embryos were further cultured in culture media for subsequent observation and analysis.

### 4.7 Karyotype identification of blastocysts

For mice, the zona pellucida of well‐developed blastocysts was removed in Acid Tyrode's Solution (pH 2.5, R23013, Yuanye Bio‐Tec, China), and then the whole embryo was placed in the lysate and stored at −20°C, and PGT‐A analysis based on NGS technology was carried out (Yikon Genomics, China).

In humans, the zona pellucida of well‐developed blastocysts was punctured by a microlaser, and then some trophoblast cells were removed by a PGT biopsy needle. The removed cells were dissolved in lysate, and PGT‐A analysis based on NGS technology was carried out (Yikon Genomics).

### 4.8 ES derivation, cell culture, and sample collection

To isolate mES cells, blastocysts were treated with Tyrode's Solution (pH 2.5, R23013, Yuanye Bio‐Tec, China) to remove the zona pellucida and plated on CF1 feeder cells in 2 µL medium.^40^ The outgrowth emerged approximately 5 days later, and the ES cell clone was picked up and digested with TrypLe Express (Gibco) and subcultured in a new gelatin‐treated well. ES cells were cultured under feeder‐free conditions from passages 3 to 21. To detect mtDNA carryover in the ES cells, 5–10 clones were randomly taken and sampled three times from each well. All clones were washed three times and then collected in a 0.2 mL tube. For oocyte and embryo collection, after removing the zona pellucida with Tyrode's Solution (Sigma), all samples were washed three times in PBS, collected in tubes containing ∼0.5 µL PBS and frozen at −20°C.

### 4.9 DNA template preparation and digital PCR detection

For DNA template preparation, samples were lysed with 50 mM NaOH (8.5 µL for oocytes, 17 µL for ES cell clones, and 85 µL for adult mouse tissue) for 30 min at 95°C and neutralized with 1 M Tris‐HCl (pH 8.0) (1.5 µL for oocytes, 3 µL for ES cell clones, and 15 µL for adult mouse tissue). To detect the mtDNA carryover ratio, locked nucleic acid probes and primers (Table S1) specific to mouse mtDNA.9348G>A and human mtDNA.8271‐8279del were designed and synthesized by Integrated DNA Technologies (Singapore). ddPCR Supermix for Probes (1863025, Bio‐Rad Laboratories, USA) was used for all experiments, with 900 nM primer, 250 nM probe, and 1 µL DNA template. The amplification product length was 85 bp. Droplets were generated using the QX200 Droplet Generator, transferred into 96‐well plates (Bio‐Rad Laboratories), sealed with a Pierceable Foil Heat Seal (Bio‐Rad Laboratories), and cycled in a C1000 Thermal Cycle (Bio‐Rad Laboratories). PCR protocol: 95°C, 10 min for 1 min; 94°C, 30 s; 60°C, 1 min for 40 cycles; 98°C, 10 min for one cycle. Droplets were read using a QX200 Droplet Reader, and data were analyzed using QuantaSoft Software (Bio‐Rad Laboratories). Each Fluorescein signal was recorded as a normal mitochondrial, and each Hexachloro‐Fluorescein signal was recorded as a mutant mitochondrial. The total number of records is the total number of mitochondria.

To detect mitochondrial residues in reconstructed human oocytes, we collected granulosa cells from donor volunteers with immature oocytes (German vehicle or metaphase I stage). Then, we sent their granulosa cells for digital PCR to determine the main mitochondrial type (WT or mtDNA.8271‐8279del/mutant) of each volunteer after 24 h. At the same time, these immature oocytes were put into G2 medium and cultured in an incubator at 37°C and 6% CO_2_ for in vitro maturation (IVM). Once the mitochondrial genotype of the volunteer granulosa cells was confirmed, the IVM‐mature oocytes of the volunteer were fertilized by ICSI. Subsequently, the ePB2T operation was performed between the WT and mutant oocytes, and the SRR operation was performed on the reconstructed oocytes. Then, once the 2PN pronucleus appeared, the whole reconstructed oocyte was sampled cell by cell and detected by digital PCR.

### 4.10 Mouse embryo surrogate transfer

Female ICR mice at 8–10 weeks of estrus were selected and caged with vasectomized male mice. The vaginal plug was examined the next morning. Female mice with vaginal plugs were selected and anesthetized. The ovary–fallopian tube complex was pulled out through the back. Then, the reconstructed and WT embryos that developed to the two‐cell stage were transferred to the fimbria of the fallopian tube. The surrogate mother mice were put into a clean cage with sufficient water and high‐fat food and fed for 21–25 days, and the birth of their offspring was observed.

### 4.11 Statistical methods

Statistical analysis was carried out using SPSS 24.0 statistical software. Continuous variables were presented as mean ± SD. The normality of continuous variables was determined using the Shapiro–Wilk test. The variables were compared by Student's *t*‐test or one‐way ANOVA with post hoc analysis (defining LSD as the method that aims to correct for multiple pairwise tests). Categorical variables were put forward in the number of cases (*n*) with percentages (%). The comparisons of rates between groups were completed by the chi‐square test. *p* < 0.05 was considered statistically significant.

## AUTHOR CONTRIBUTIONS

Q.L. and Y.K. conceived and designed the experiments. W.L., X.L., K.L., R.C., and H.G. performed the experiments. W.L., X.L., K.L., Z.Y., J.S., and L.Z. established the research methods. R.C., H.G., Y.W., S.Z., H.G., and Y.W. collected the human samples. M.M., Y.X., L.C., W.Y., and C.C. collected the mouse samples. W.L., X.L., and K.L. analyzed the data. W.L., X.L., and Q.L. wrote the paper. All authors approved the final manuscript.

## CONFLICT OF INTEREST STATEMENT

The authors declare no conflicts of interest.

## ETHICS STATEMENT

All animal experiments were conducted in accordance with the Guide for the Care and Use of Animals for Research Purposes. The experiments for mouse embryos were approved by the Institutional Animal Care and Use Committee and Internal Review Board of Shanghai Jiao Tong University School of Medicine (approval number: HKDL [2018]228). All human experiments were approved by the Institutional Review Board of Shanghai Jiao Tong University School of Medicine (approval number: SH9H‐2018‐T57‐1), and informed consent was obtained from all of the participants.

## Supporting information



Supporting Information

Movie S1

Movie S2

Movie S3

## Data Availability

All the data supporting the conclusions of this study are included within the article and Supporting Information. All the other data are available upon request to the corresponding author.
