# Supplementary material for: Earlier second polar body transfer and further mitochondrial carryover removal for potential mitochondrial replacement therapy
Source: MedComm (2020). 2023 May 8;4(3):e217. doi: 10.1002/mco2.217 (PMC10167372; doi:10.1002/mco2.217)
Supplement: Supplementary file 1 — Supporting Information [file MCO2-4-e217-s004.docx]

**Title:**

**Additional mitochondrial carryover removal in second polar body transfer for potential mitochondrial replacement therapy**

**Running Title:**

**Additional mitochondrial carryover removal in second polar body transfer**

**Authors:** Wenzhi Li#, Xiaoyu Liao#, Kaibo Lin#, Renfei Cai#, Haiyan Guo#, Meng Ma, Yao Wang, Yating Xie, Shaozhen Zhang, Zhiguang Yan, Jiqiang Si, Hongyuan Gao, Leiwen Zhao, Li Chen, Weina Yu, Chen Chen, Yun Wang, Yanping Kuang*, Qifeng Lyu*

1. Department of Assisted Reproduction, Shanghai Ninth People's Hospital, Shanghai Jiaotong University School of Medicine, Shanghai, People's Republic of China

***Correspondence address:** Department of Assisted Reproduction, Shanghai 9th People’s Hospital, Shanghai Jiao Tong University School of Medicine, No. 639 Zhizaoju Road, Shanghai 200011, China. E-mail: kuangyanp@126.com (Y.K.), lyuqifeng@126.com (Q.L.)

**† These authors have contributed equally to this work.**

**Funding:** The National Natural Science Foundation of China (81871163), National Key Research and Development Program of China (2018YFC1003000) and the National Natural Science Foundation of China (81901478, 32000599).

**Supplementary materials**

**Figure S1**

**
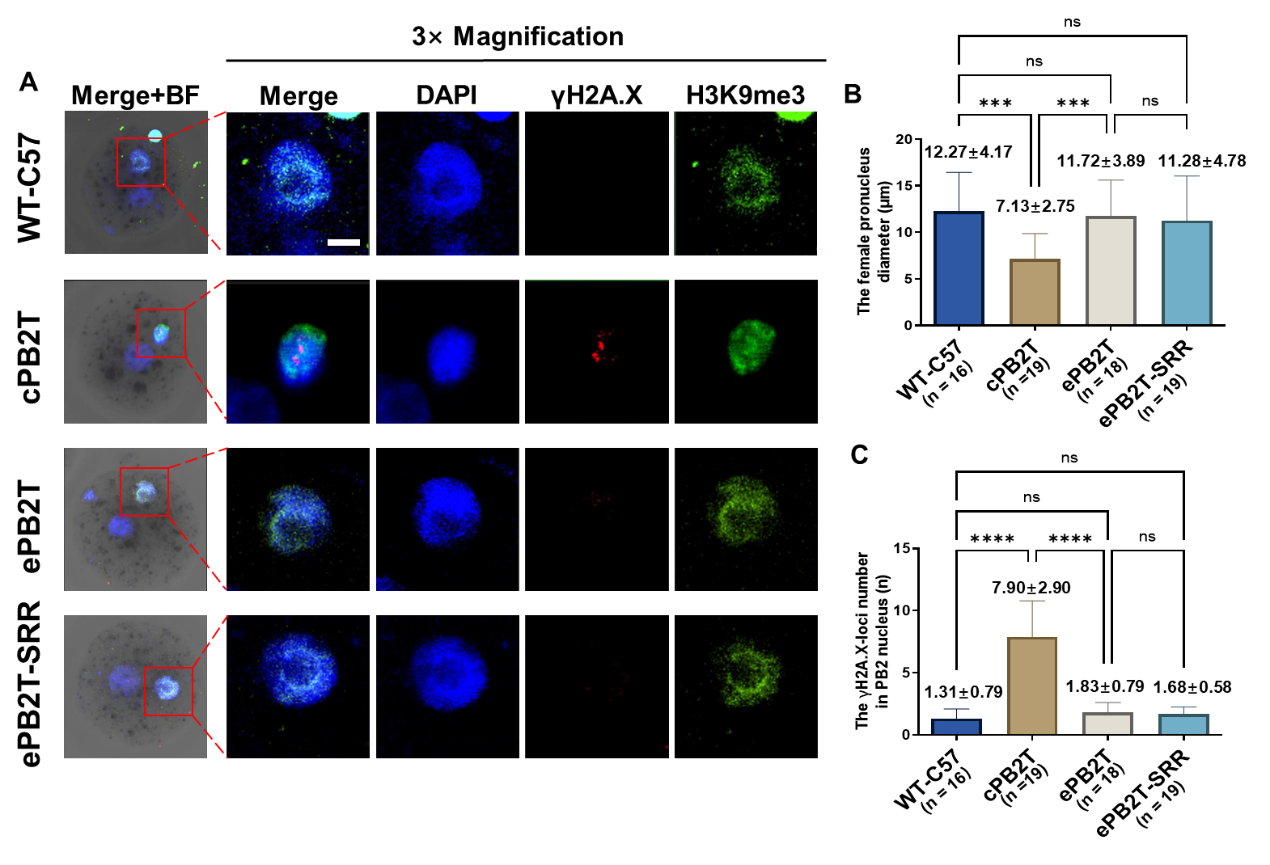
**

**Figure S1. Trend of DNA damage in the female pronucleus at late PN stage.**

1. Representative images of DNA damage (γH2A. X based) in PB2 of zygotic stage embryos with different PB2 extrusion times in the WT-C57, cPB2T, ePB2T and ePB2T-SRR groups. γH2A.X (red), H3K9me3 (green, used to indicate the female pronucleus), and DAPI (blue). The red box indicates the female pronucleus nucleus, and there is a 3× magnification for female pronucleus. Scale bars, 7.5 μm.
2. Quantification of the female pronucleus diameter (μm, Y-axis) in the WT-C57 (n=16), cPB2T (n=19), ePB2T (n=18) and ePB2T-SRR (n=19) groups (X-axis). The bar and whiskers represent the mean and SD. One-way ANOVA demonstrated significant differences among the four groups. LSD multiple comparisons tests were performed to compare the significance among the groups, ***P < 0.001.
3. Quantification of the PB2 nucleus γH2A. X-loci number (Y-axis) in the WT-C57 (n=16), cPB2T (n=19), ePB2T (n=18) and ePB2T-SRR (n=19) groups (X-axis). The bar and whiskers represent the mean and SD. One-way ANOVA demonstrated significant differences among the four groups. LSD multiple comparisons tests were performed to compare the significance among the groups, ****P < 0.0001.

**Figure S2**

**
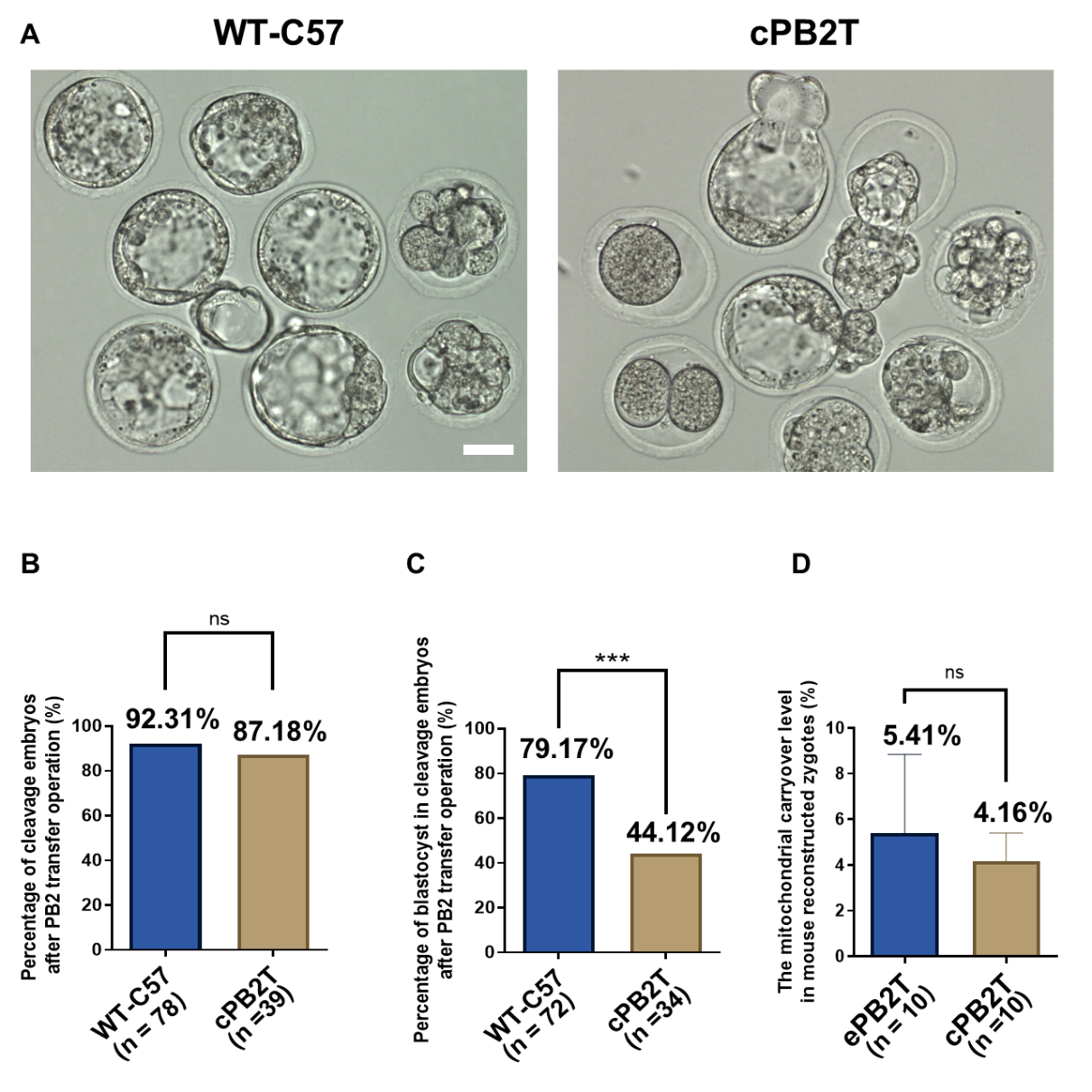
**

**Figure S2. Developmental potential of reconstructed embryo in the cPB2T scheme.**

1. Representative images of blastocyst formation in the WT-C57 and cPB2T groups. Scale bars, 50 μm.
2. Quantification of the embryo cleavage rate (%, Y-axis) in the WT-C57 (n=78) and cPB2T (n=39) groups (X-axis). Χ²-test, P > 0.05.
3. Quantification of the blastocyst formation rate (%, Y-axis) in the WT-C57 (n=72) and cPB2T (n=34) (X-axis). Χ²-test, ***P < 0.001.
4. Quantification of the mouse reconstructed zygote mitochondrial carryover level (mitochondrial carryover number/total mitochondria number, %, Y-axis) in the ePB2T (n=10) and cPB2T (n=10) groups (X-axis). The bar and whiskers represent the mean and SD. t-test were performed to compare significance between groups, P > 0.05.

**Figure S3**

**
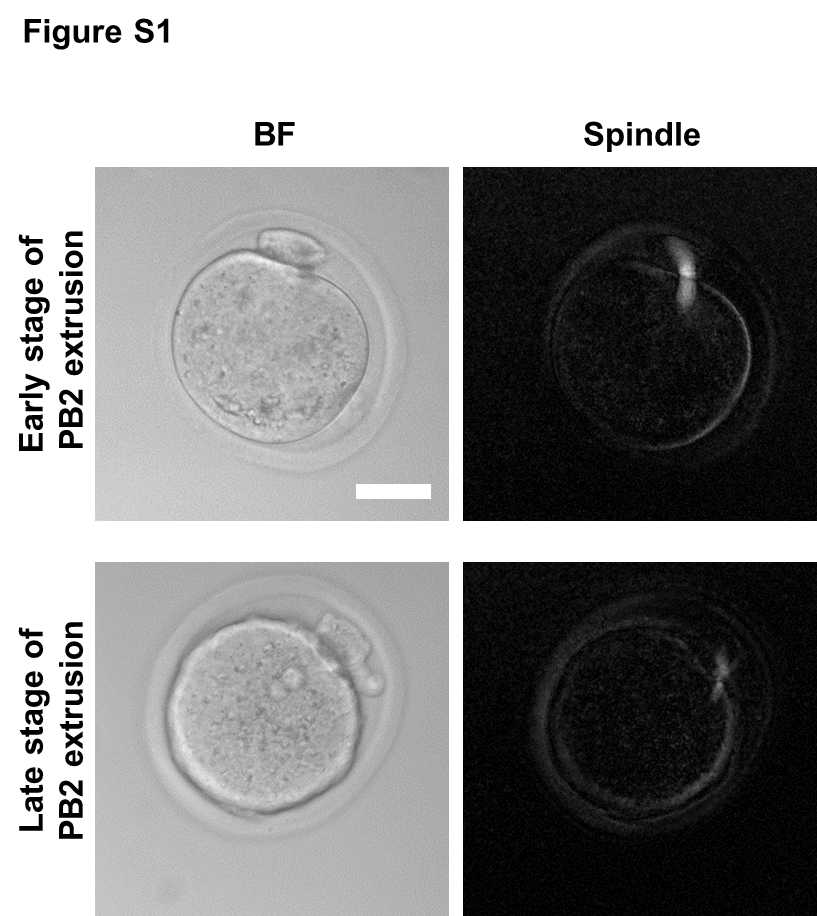
**

**Figure S3.** **Spindle observation in the early and late stages of PB2 extrusion.**

Representative images of Spindle strength in the early and late stages of PB2 extrusion. Bright field (left), Spindle observation (right). Scale bars, 20 μm.

**Figure S4**

**
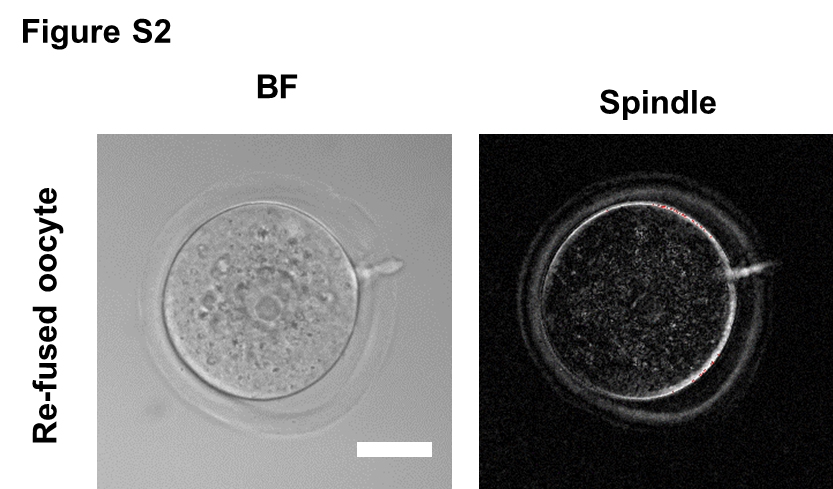
**

**Figure S4.** **PB2 with SP reintegrated into enucleated oocyte.**

Representative image of the re-fused oocyte with SP protrusion. Scale bars, 20 μm.

**Figure S5**

**
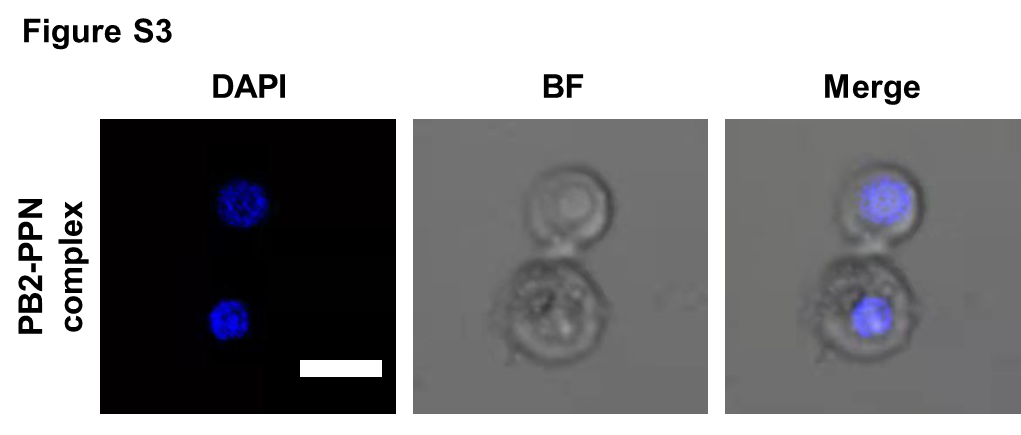
**

**Figure S5.** **Major genetic material distribution of the PB2-FPPN complex obtained in the ePB2T scheme.**

Representative image of the major genetic material distribution in the PB2-FPPN complex, DAPI (blue). Scale bars, 20 μm.

**Figure S6**

**
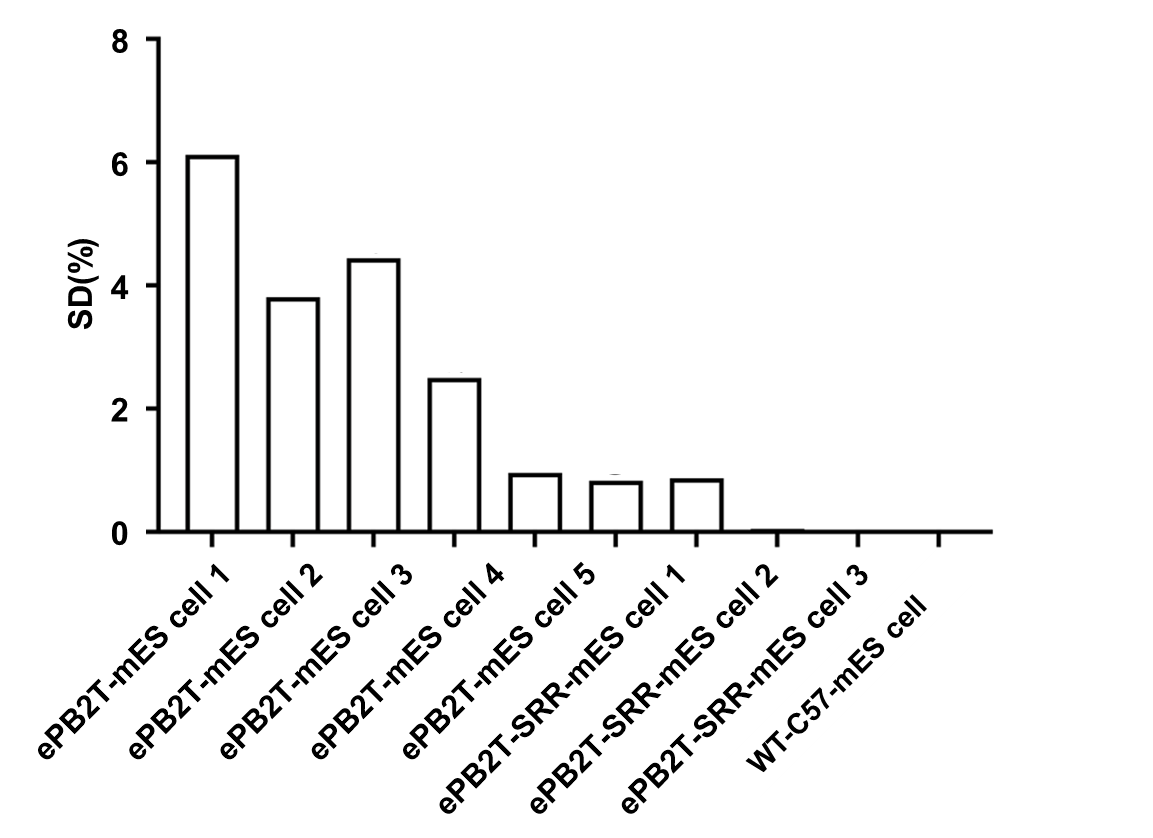
**

**Figure S6.** **Mitochondrial heterogeneity variation of mES cell samples.**

The level of mitochondrial genetic drift (based on SD value) in mES cell lines obtained from reconstructed embryos with ePB2T or ePB2T-SRR scheme.

**Figure S7**

**
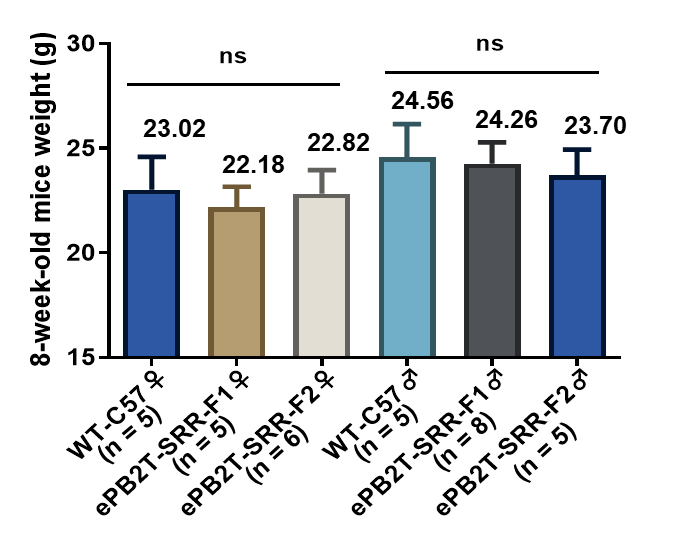
**

**Figure S7 The adult weights (8-week-old) of WT-C57 and reconstructed F1/F2 mice.**

Quantification of the 8-week-old mouse weight (g, Y-axis) in ♀WT-C57 (n=5), ePB2T-SRR-♀F1 (n=5), ePB2T-SRR-♀F2 (n=6), ♂WT-C57 (n=5), ePB2T-SRR-♂F1 (n=8) and ePB2T-SRR-♂F2 (n=5) groups (X-axis). The bar and whiskers represent the mean and SD. One-way ANOVA demonstrated significant differences among the three groups, LSD multiple comparisons tests were performed to compare the significance among the groups, P > 0.05.

**Figure S8**

**
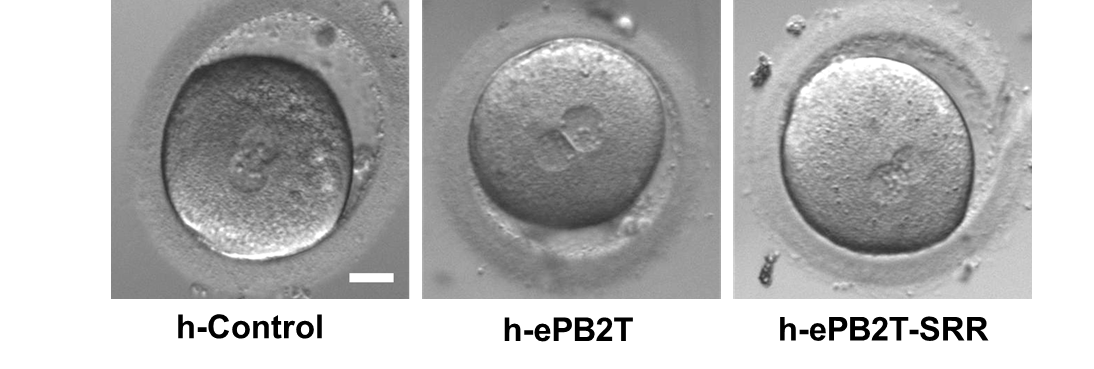
**

**Figure S8.** **Representative images of the reconstructed human 2PN zygotes.**

Representative images of the reconstructed human 2PN zygotes in the h-Control, h-ePB2T and h-ePB2T-SRR groups. Scale bars, 30 μm.

**Figure S9**

**Figure S9.** **Normal karyotype rate of blastocyst based on cleavage embryo number.**

Quantification of the abnormal karyotype blastocyst based on cleavage embryo number (%, Y-axis) in the Control (n=72), ePB2T (n=36) and ePB2T-SRR (n=44) groups (X-axis). Χ²-test, *P ＜0.05.

**Table S1 Digital PCR primer sequence for mitochondrial counting.**

| Primer Name | Species | Strain | Sequence |
| --- | --- | --- | --- |
| mPrimer-F | Mouse | N.A | CGC AGC ATG ATA CTG ACA T |
| mPrimer-R | Mouse | N.A | ACT AAG GGA GTA AGA TCC TCA T |
| mProbe-C57 | Mouse | C57BL/6 | AA+GTCAG+A+C+TA+C+GT (FAM) |
| mProbe-BALB | Mouse | BALB/c | AG+TCAG+A+T+TA+CGT+CT (HEX) |
| hPrimer-F | Human | N.A | AAACCACAGTTTCATGCCC |
| hPrimer-R | Human | N.A | AAGAGGTGTTGGTTCTCTTAATCT |
| hProbe-WT | Human | Wild type | CTC+T+A+CC+C+CC (FAM) |
| hProbe-MU | Human | Mutant | AG+CACCCCCTCTA+GAG (HEX) |

**Table S2 Results of mouse blastocyst karyotype based on PGT-A detection.**

| Sample Name | Group | karyotype | Conclusion |
| --- | --- | --- | --- |
| mB-1 | ePB2T-SRR | 40,XX | Normal |
| mB-2 | ePB2T-SRR | 40,XX,  -15(×1,mos,~37%) | Chimeric |
| mB-3 | ePB2T-SRR | 40,XY | Normal |
| mB-4 | ePB2T-SRR | 40,XX | Normal |
| mB-5 | ePB2T-SRR | 40,XY | Normal |
| mB-6 | ePB2T-SRR | 40,XY | Normal |
| mB-7 | ePB2T-SRR | 41,XY,  +17(×3) | Abnormal |
| mB-8 | ePB2T-SRR | 40,XY | Normal |
| mB-9 | ePB2T-SRR | 40,XY | Normal |
| mB-10 | ePB2T-SRR | 40,XX | Normal |
| mB-11 | ePB2T-SRR | 40,XY | Normal |
| mB-12 | ePB2T-SRR | 40,XY | Normal |
| mB-13 | ePB2T-SRR | 39,XY,  -18(×1) | Abnormal |
| mB-14 | ePB2T-SRR | 40,XY | Normal |
| mB-15 | ePB2T-SRR | 40,XY | Normal |
| mB-16 | ePB2T-SRR | 40,XX | Normal |
| mB-17 | ePB2T-SRR | 40,XY | Normal |
| mB-18 | ePB2T-SRR | 40,XX | Normal |
| mB-19 | ePB2T-SRR | 40,XY | Normal |
| mB-20 | ePB2T-SRR | 40,XX | Normal |
| mB-21 | ePB2T-SRR | 40,XX,  +1(×3,mos,~35%) | Chimeric |
| mB-22 | ePB2T-SRR | 40,XY | Normal |
| mB-23 | ePB2T-SRR | 40,XY | Normal |
| mB-24 | ePB2T-SRR | 40,XX | Normal |
| mB-25 | ePB2T | 40,XX | Normal |
| mB-26 | ePB2T | 40,XY,  -9q(qA4→qF4,~96Mb,  1,mos,~36%) | Chimeric |
| mB-27 | ePB2T | 40,XX,  -3q(qB→qE3,~30Mb,×1) | Abnormal |
| mB-28 | ePB2T | 40,XY | Normal |
| mB-29 | ePB2T | 40,XY | Normal |
| mB-30 | ePB2T | 40,XY | Normal |
| mB-31 | ePB2T | 40,XY | Normal |
| mB-32 | ePB2T | 40,XY | Normal |
| mB-33 | ePB2T | 40,XX | Normal |
| mB-34 | ePB2T | 40,XY | Normal |
| mB-35 | ePB2T | 40,XY | Normal |
| mB-36 | ePB2T | 40,XX | Normal |
| mB-37 | ePB2T | 40,XY | Normal |
| mB-38 | ePB2T | 42,XX,  +1(×3),+5(×3) | Abnormal |
| mB-39 | ePB2T | 41,XY,  +15(×3) | Abnormal |
| mB-40 | ePB2T | Multiple abnormalities | Abnormal |
| mB-41 | ePB2T | 40,XY | Normal |
| mB-42 | ePB2T | 40,XY | Normal |
| mB-43 | ePB2T | 40,XX | Normal |
| mB-44 | Control | 40,XY | Normal |
| mB-45 | Control | 40,XX | Normal |
| mB-47 | Control | 40,XX,  -6q(qB3→qD3,~34M,×1,mos,~40%) | Chimeric |
| mB-48 | Control | 40,XY | Normal |
| mB-49 | Control | 40,XY | Normal |
| mB-50 | Control | 40,XX | Normal |
| mB-51 | Control | 40,XY | Normal |
| mB-52 | Control | 40,XX | Normal |
| mB-53 | Control | 39,XY,  -1(×1) | Abnormal |
| mB-54 | Control | 40,XY | Normal |
| mB-55 | Control | 40,XY | Normal |
| mB-56 | Control | 40,XX | Normal |
| mB-57 | Control | 40,XY,  -3(×3,mos,~32%) | Chimeric |
| mB-58 | Control | 40,XX | Normal |
| mB-59 | Control | 40,XY | Normal |
| mB-60 | Control | 40,XY | Normal |

**Table S3 Results of F1 and F2 generation mice karyotype based on PGT-A detection.**

| Sample Name | Group | Gender | karyotype | Conclusion |
| --- | --- | --- | --- | --- |
| mF1-1 | ePB2T-SRR-F1 | Female | 40,XX | Normal |
| mF1-2 | ePB2T-SRR-F1 | Female | 40,XX | Normal |
| mF1-3 | ePB2T-SRR-F1 | Female | 40,XX | Normal |
| mF1-4 | ePB2T-SRR-F1 | Female | 40,XX | Normal |
| mF1-5 | ePB2T-SRR-F1 | Female | 40,XX | Normal |
| mF1-6 | ePB2T-SRR-F1 | Male | 40,XY | Normal |
| mF1-7 | ePB2T-SRR-F1 | Male | 40,XY | Normal |
| mF1-8 | ePB2T-SRR-F1 | Male | 40,XY | Normal |
| mF1-9 | ePB2T-SRR-F1 | Male | 40,XY | Normal |
| mF1-10 | ePB2T-SRR-F1 | Male | 40,XY | Normal |
| mF1-11 | ePB2T-SRR-F1 | Male | 40,XY | Normal |
| mF1-12 | ePB2T-SRR-F1 | Male | 40,XY | Normal |
| mF1-13 | ePB2T-SRR-F1 | Male | 40,XY | Normal |
| mF2-1 | ePB2T-SRR-F2 | Female | 40,XX | Normal |
| mF2-2 | ePB2T-SRR-F2 | Female | 40,XX | Normal |
| mF2-3 | ePB2T-SRR-F2 | Female | 40,XX | Normal |
| mF2-4 | ePB2T-SRR-F2 | Female | 40,XX | Normal |
| mF2-5 | ePB2T-SRR-F2 | Female | 40,XX | Normal |
| mF2-6 | ePB2T-SRR-F2 | Female | 40,XX | Normal |
| mF2-7 | ePB2T-SRR-F2 | Male | 40,XY | Normal |
| mF2-8 | ePB2T-SRR-F2 | Male | 40,XY | Normal |
| mF2-9 | ePB2T-SRR-F2 | Male | 40,XY | Normal |
| mF2-10 | ePB2T-SRR-F2 | Male | 40,XY | Normal |
| mF2-11 | ePB2T-SRR-F2 | Male | 40,XY | Normal |

**Table S4 Results of human blastocyst karyotype based on PGT-A detection.**

| Sample Name | Group | karyotype | State |
| --- | --- | --- | --- |
| hB-1 | h-ePB2T-SRR | 46,XN | Normal |
| hB-2 | h-ePB2T-SRR | 46,XN | Normal |
| hB-3 | h-ePB2T-SRR | 46,XN | Normal |
| hB-4 | h-ePB2T-SRR | 46,XN | Normal |
| hB-5 | h-ePB2T-SRR | 46,XN | Normal |
| hB-6 | h-ePB2T-SRR | 46,XN,  -7p(×1),  -7q(q11.21→q21.13,~30Mb,×1),  -7q(q21.2→q31.2,~24Mb,×1,mos,~57%),  -7q(q31.32→q36.3,~37Mb,×1,mos,~54%) | Abnormal |
| hB-7 | h-ePB2T-SRR | 46,XN | Normal |
| hB-8 | h-ePB2T-SRR | 46,XN | Normal |
| hB-9 | h-Control | 46,XN,  -1p(p36.32→p36.13,~17Mb,×1,mos,~66%),  -1p(p36.12→p12,~99Mb,×1),-1q(×1) | Abnormal |
| hB-10 | h-Control | 46,XN | Normal |
| hB-11 | h-Control | 46,XX,  -Xp(p11.23→p11.21,~10Mb,×1,mos,~34%),  -Xq(×1,mos,~34%) | Chimeric |
| hB-12 | h-Control | 46,XN | Normal |
| hB-13 | h-Control | 46,XN,  -4p(×1,mos,~32%) | Chimeric |
| hB-14 | h-Control | 46,XN | Normal |
| hB-15 | h-Control | 46,XN | Normal |
| hB-16 | h-Control | 46,XN | Normal |
| hB-17 | h-Control | 46,XN | Normal |
| hB-18 | h-Control | 46,XN | Normal |
| hB-19 | h-Control | 46,XN | Normal |
| hB-20 | h-Control | 46,XN | Normal |
| hB-21 | h-Control | 46,XN | Normal |
| hB-22 | h-Control | 46,XN,  -1p(p36.32→p36.22,~6.0Mb,×1),  -2q(q14.1→q37.3,~126Mb,×1,mos,~59%) | Abnormal |
| hB-23 | h-Control | 46,XN | Normal |
| hB-24 | h-Control | 46,XN,  -18q(q21.2→q23,~25Mb,×1,mos,~31%) | Chimeric |
| hB-25 | h-Control | 47,XN,  +16(×3) | Abnormal |
| Hb-26 | h-Control | 46,XN | Normal |

**Table S5 Process of ePB2T-SRR scheme operation**

| Steps | Operation details |
| --- | --- |
| a | Position the PB1 of the oocyte at 12 o'clock, then perform ICSI. |
| b | Punch through the zonal pellucida with a microlaser (ZILOS-tk, Hamilton Thorne, USA) at 1-2 o'clock position, then remove the PB1 of human or mice by ICSI needle (most mouse PB1s have been naturally apoptotic). |
| c | c1, Transfer the PB2 extruded oocytes to operating medium for 5–10 minutes;  c2, Hold the oocyte and place the PB2 of the oocyte at 1 o'clock position;  c3, Further expand the gap on the 2-3 o'clock position of the zonal pellucida (If necessary);  c4, PB2 and a certain volume (~1.5× PB2 volume for mouse, ~2× PB2 volume for human) of cytoplasm (containing female pre-pronucleus, FPPNs) connected to PB2 through the spindle were carefully removed from oocytes by a 15 μm-flat biopsy needle under a spindle observation microscope. |
| d | Shake off the FPPN from the PB2-spindle complex carefully in a modified viscous medium (Contain Polyvinylpyrrolidone/PVP Solution, 90121, Fujifilm, Japan); |
| e | Turn the direction of the PB2-spindle complex and make half of the cytoplasm of PB2 contact inactivated Sendai virus (1:5 Dilution, 40s for mouse, 60s for human). |
| f | Transfer the PB2-spindle complex to a FPPN-enucleated oocyte, and push the complex out to make it full contact with the oocyte membrane for 2 minutes. PB2 will fuse with the oocyte within half an hour, but the spindle will remain at the fusion site. |
| g | A certain time after fusion (1-hour for mouse, 2-hour for human), a 12 μm flat biopsy needle was used to suck the cytoplasm (0.5~1× PB2 volume for mouse, ~1× PB2 volume for human) with the spindle as the fusion site marker (Avoid the initially apparent female pronucleus), and the sucked cytoplasm was carefully separated from the oocyte. |
| h | Then, these reconstructed embryos were further cultured in culture media for subsequent observation and analysis. |
| *** Since the heat generated during microlaser drilling may damage the genetic material of spindle or PB2, great care should be taken when punching the zonal pellucida: Control the laser intensity not to exceed 150 μs and ensure that there is a distance of 1-2 o'clock position between the genetic material and the punching position (e.g., step b and step c3).  *** When patients (and donors) have a large number of oocytes, each batch of ICSI is controlled at about 2-3 pairs, and the interval between each batch of ICSI is about 1 hour. | |

**Movie S1. Representative movie of female pronucleus and originally carried mitochondria migration in mouse reconstructed zygotes.**

**Movie S2. Representative movie of ePB2T-SRR scheme operation in mouse oocytes.**

**Movie S3. Representative movie of ePB2T-SRR scheme operation in human oocytes.**
